# Comparison of mifepristone plus misoprostol with misoprostol alone for first trimester medical abortion: A systematic review and meta-analysis

**DOI:** 10.3389/fgwh.2023.1112392

**Published:** 2023-03-06

**Authors:** Tariku Shimels, Melsew Getnet, Mensur Shafie, Lemi Belay

**Affiliations:** ^1^ Research Directorate, St. Paul’s Hospital Millennium Medical College, Addis Ababa, Ethiopia; ^2^ Department of Pharmacology, St. Paul’s Hospital Millennium Medical College, Addis Ababa, Ethiopia; ^3^ Department of Obstetrics and Gynaecology, St. Paul’s Hospital Millennium Medical College, Addis Ababa, Ethiopia

**Keywords:** first trimester, mifepristone plus misoprostol, medical abortion, misoprostol alone, systematic review and meta-analysis

## Abstract

**Objective:**

To compare mifepristone plus a misoprostol-combined regimen with misoprostol alone in the medical abortion of first trimester pregnancy.

**Methods:**

An internet-based search of available literature was performed using text words contained in titles and abstracts. PubMed/Medline, Cochrane CENTRAL, EMBASE, and Google scholar were used to locate English-based articles published until December 2021. Studies fulfilling the inclusion criteria were selected, appraised, and assessed for methodological quality. The included studies were pooled for meta-analysis, and the results were presented in risk ratio at a 95% confidence interval.

**Findings:**

Nine studies comprising 2,052 participants (1,035 intervention and 1,017 controls) were considered. Primary endpoints were complete expulsion, incomplete expulsion, missed abortion, and ongoing pregnancy. The intervention was found to more likely induce complete expulsion irrespective of gestational age (RR: 1.19; 95% CI: 1.14–1.25). The administration of misoprostol 800 mcg after 24 h of mifepristone pre-treatment in the intervention group more likely induced complete expulsion (RR: 1.23; 95% CI: 1.17–1.30) than after 48 h. The intervention group was also more likely to experience complete expulsion when misoprostol was used either vaginally (RR: 1.16; 95% CI: 1.09–1.17) or buccally (RR: 1.23; 95% CI: 1.16–1.30). The intervention was more effective in the subgroup with a negative foetal heartbeat at reducing incomplete abortion (RR: 0.45; 95% CI: 0.26–0.78) compared with the control group. The intervention more likely reduced both missed abortion (RR: 0.21; 95% CI: 0.08–0.91) and ongoing pregnancy (RR: 0.12; 95% CI: 0.05–0.26). Fever was less likely to be reported (RR: 0.78; 95% CI: 0.12–0.89), whereas the subjective experience of bleeding was more likely to be encountered (RR: 1.31; 95% CI: 1.13–1.53) by the intervention group.

**Conclusion:**

The review strengthened the theory that a combined mifepristone and misoprostol regimen can be an effective medical management for inducing abortions during first trimester pregnancy in all contexts. Specifically, there is a high-level certainty of evidence on complete expulsion during the early stage and its ability to reduce both missed and ongoing pregnancies.

**Systematic Review Registration:**

https://www.crd.york.ac.uk/prospero/display_record.php?ID=CRD42019134213, identifier CRD42019134213.

## Introduction

Abortion is a medical phenomenon that requires either a drug- or a non-drug-based intervention (1). The reasons for seeking abortion services may vary among different groups. A significant number of pregnant women appear to visit health facilities for emergency management of induced abortion (2). While those women with unwanted pregnancy and having an intention to stop at early weeks of gestation is one inevitable characteristic of this group, attendance following an intrauterine foetal death or viability failure, due to various causes, is frequently mentioned (3–5). Numerous studies indicate that using medications, especially mifepristone and misoprostol, is preferred over aspiration or curettage interventions for terminating a first trimester pregnancy (6, 7) or evacuating an early pregnancy failure (8). The drugs can be self-administered with a maximal success rate compared with manual aspirations (8–10). According to a study in 2012, approximately 75% of healthcare providers in Canada and 98% of providers in the United States offered medication abortion to people younger than 18 years of age (6). A report also showed that medication abortion accounted approximately 45% of the abortions in the United States in 2022 (11), and out of the legally induced abortions reported to the Centers for Disease Control and Prevention in 2020, 51.0% were early-stage ones (12).

Endogenous substances with the property of uterine contractility include prostaglandins (PGE2 and PGF2a) and their synthetic analogues (gemeprost, sulprostone, meteneprost, and misoprostol), cytotoxic drugs such as methotrexate, anti-progesterone mifepristone, and aromatic organic compounds such as ethacridine lactate (13, 14). It is widely accepted that a remarkable possibility of attaining a complete expulsion of conceptus tissue occurs when prostaglandin analogues and mifepristone are used together (15, 16). The introduction of these agents in maternal healthcare has also resulted in a breakthrough in preventing premature mortality and pregnancy-related maternal complications (17).

The effect of prostaglandins alone and combined agents in first trimester pregnancy termination was evaluated in a systematic review by Kulier et al. (18). Four out of five studies included in the review compared combinations other than mifepristone and misoprostol against misoprostol alone on successful abortion and side effects. To examine more outcomes and include studies after 2004 was sought as an additional justification for this review. The two regimens have important pharmacokinetic and pharmacodynamic properties, making them drugs of choice in maternal healthcare and family planning programmes. Among all prostaglandin analogues that contract the uterus and ripen the cervix, misoprostol is the most widely used agent that is also orally active, stable at room temperature, and relatively inexpensive (19, 20). In addition, it is well absorbed following oral, vaginal, buccal, or sublingual administration and has a proven safety record (20). Advances in reproductive health and gynaecology practices have enabled the administration of mifepristone, a progesterone receptor antagonist, prior to misoprostol, to attain effective termination of pregnancy (21). This drug substantially blocks the P receptors (progesterone receptors) in the placenta, resulting in the cessation of uterine implantation (22). A combination of mifepristone with misoprostol, even at a low dose, is highly effective and acceptable as a self-administered abortifacient often recommended as the preferred combination regimen (22–24).

Original clinical trials (23–25) and reviews (26, 27) showed that the combination regimen of mifepristone and misoprostol resulted in a higher success rate compared with the misoprostol alone in second trimester abortions. On the other hand, a systematic review conducted to determine the effects of mifepristone during third trimester cervical ripening concluded to have inadequate evidence for suggesting the drug for labour induction (28). A thorough investigation into whether the termination of first trimester pregnancy with mifepristone followed by misoprostol would produce a better outcome when compared with misoprostol alone remains uncertain. In addition, the fact that least safe practices of abortion procedures are rising from 1% in developed countries to 54% in developing countries, along with the ever-growing prevalence of very early trimester abortions (<9 weeks of gestation) worldwide, with most groups at risk being younger women (29), calls for collecting a rich source of information and making a precise level of estimate on the effect of the two drugs in this population.

The rate of successful abortion was also reported to vary with the timing of subsequent misoprostol administration following mifepristone (30). The systematic reviews conducted so far have significant variations in terms of the designs employed, drugs considered, target population factors, as well as statistical measures applied by original studies, consequently ending with diverse conclusions (31, 32). This again raises a question whether the conjugate result really assures that what is claimed in certain controlled trials (23, 30, 33–38) holds a consistent trend strength and direction of effect in extended weeks of gestation. Apart from the effect on terminated live tissue, the expulsion rate of a dead embryo prior to drug administration would be one factor requiring evaluation.

The objective of this systematic review is to compare the mifepristone plus misoprostol regimen with misoprostol alone in the medical abortion of first trimester pregnancy on the basis of randomized or quasi-randomized control trials conducted at different times until December 2021.

## Review question(s)

This review throws up the question: What is the effectiveness of mifepristone plus misoprostol compared with misoprostol alone for inducing abortion in the first trimester of pregnancy. More specifically, it attempts to evaluate and compare the incidence of complete abortion and potential complications, namely, incomplete abortion, missed abortion, and ongoing pregnancies as well as side effects following the administration of the respective regimens in both populations.

## Inclusion criteria

### Participants

The review considered studies that included pregnant women with live or dead foetus during the first trimester (≤12 weeks of gestation) who visited health facilities seeking induced medical abortion. Studies that involved pregnant women who received additional means of interventions along with drugs, those who did not come under the purview of the defined trimester, or those presented with amniotic sac out of the uterus were excluded.

### Intervention(s)

This review considered controlled clinical trials with randomized study populations to receive mifepristone plus misoprostol as an intervention group for first trimester abortion. Misoprostol could be administered at least 24 h apart from mifepristone by any route. When necessary, additional doses of misoprostol might be considered.

### Comparator(s)

Populations that have been assigned to receive the misoprostol-alone regimen as an alternative means of first trimester medical abortion were considered comparators. The drug could be administered after or followed by placebo and 3–48 h apart between subsequent doses. Frequency may depend on unit doses and last at least until the third day *via* any route.

### Outcomes

This review considered incidence of the following outcomes: complete expulsion or abortion, incomplete abortion, missed abortion or miscarriage, and ongoing or continuing pregnancy confirmed by ultrasound sonography and expert opinion. In addition, incidence of any other form of complications and side effects following medication administration was evaluated. These outcomes were measured by using risk ratio (RR).

### Types of studies

All controlled clinical trials with true or quasi randomization were planned for inclusion. As the problem in question is best addressed through controlled designs, such studies published right from database inception to December 2021 were considered in the review. Because of language barriers, articles published in a language other than English were not eligible.

## Methods

This systematic review was conducted in accordance with the Joanna Briggs Institute (JBI) methodology for systematic reviews of effectiveness evidence (39).

### Search strategy

The search strategy was aimed to locate both published and unpublished studies. An initial limited search of Medline and Cochrane CENTRAL was undertaken to identify articles on the topic. The text words contained in the titles and abstracts of relevant articles and the index terms used to describe the articles were used to develop a full search strategy for PubMed/Medline (Supplementary Appendix SI), Cochrane CENTRAL (Supplementary Appendix SI), and EMBASE (Ovid) (Supplementary Appendix SI), WHO Trial Registration dataset, and Google Scholar. The search strategy that covers all identified keywords and index terms was adapted for each included information source. The reference list of all studies selected for critical appraisal was screened for additional studies.

### Information sources

An electronic search of various databases or digital libraries such as PubMed, EMBASE, and Cochrane CENTRAL was done for published reports. Grey literature sources as Google Scholar and the WHO international clinical trial registry platform were included as a source log.

### Study selection

Following the search, all identified citations were collated and uploaded into EndNote and duplicates were removed. Titles and abstracts were then screened by two independent reviewers for assessment against the inclusion criteria for the review. Potentially relevant studies were retrieved in full and their citation details imported into the Joanna Briggs Institute System for the Unified Management, Assessment and Review of Information (JBI SUMARI) (Joanna Briggs Institute, Adelaide, Australia) (40). The full text of selected citations was assessed in detail against the inclusion criteria by two independent reviewers. Reasons for exclusion of full text studies that did not meet the inclusion criteria were recorded and reported in the systematic review. Any disagreements that arose between the reviewers at each stage of the study selection process were resolved through discussion or with a third reviewer. The results of the search were reported in full in the final systematic review and presented in a Preferred Reporting Items for Systematic Reviews and Meta-analyses (PRISMA) flow diagram (41).

### Assessment of methodological quality

Eligible studies were critically appraised by two independent reviewers (TS and LB) at the study level for methodological quality in the review using standardized critical appraisal instruments from the Joanna Briggs Institute for experimental studies (40). Any disagreements that arose were resolved through discussion or with a third reviewer. The results of critical appraisal were reported in narrative form and in a table (Table 1).

**Table 1:** Methodological quality assessment of included studies.

Citation	Q1	Q2	Q3	Q4	Q5	Q6	Q7	Q8	Q9	Q10	Q11	Q12	Q13
Blum et al. (38)	Y	Y	Y	Y	Y	Y	Y	Y	Y	Y	Y	Y	Y
Chawdhary et al. (36)	N	N	U	N	N	Y	Y	Y	Y	Y	Y	Y	N
Dahiya et al. (37)	U	U	Y	U	N	Y	Y	Y	Y	Y	Y	Y	U
Dalenda et al. (32)	N	N	Y	N	N	Y	Y	Y	Y	Y	Y	Y	N
Fekih et al. (50)	Y	Y	Y	Y	N	Y	Y	Y	Y	Y	Y	Y	Y
Jain et al. (35)	Y	Y	Y	Y	Y	Y	Y	Y	Y	Y	Y	Y	Y
Ngoc et al. (23)	Y	Y	Y	Y	Y	Y	Y	Y	Y	Y	Y	Y	Y
Schreiberet al. (30)	Y	Y	Y	N	N	Y	Y	Y	Y	Y	Y	Y	Y
Stockheim et al. (34)	Y	Y	Y	N	N	U	Y	Y	Y	Y	Y	Y	Y
%	66.66	66.66	88.88	44.44	33.33	88.88	100.0	100.0	100.0	100.0	100.0	100.0	66.66

Q1: Was true randomization used for assignment of participants to treatment groups? Q2: Was allocation to treatment groups concealed? Q3: Were treatment groups similar at the baseline? Q4: Were participants blind to treatment assignment? Q5: Were those delivering treatment blind to treatment assignment? Q6: Were outcome assessors blind to treatment assignment? Q7: Were treatment groups treated identically other than the intervention of interest? Q8: Was follow-up complete and if not, were differences between groups in terms of their follow-up adequately described and analyzed? Q9: Were participants analyzed in the groups to which they were randomized? Q10: Were outcomes measured in the same way for treatment groups? Q11: Were outcomes measured in a reliable way? Q12: Was appropriate statistical analysis used? Q13: Was the trial design appropriate, and were any deviations from the standard RCT design (individual randomization, parallel groups) accounted for in the conduct and analysis of the trial?

### Data extraction

Data were extracted from studies included in the review by two independent reviewers (TS and LB) using the standardized data extraction tool from the JBI database (40). The data extracted that included specific details about the populations, study methods, interventions, and outcomes of significance to the review objective indicated the specific details. Any disagreements that arose between the reviewers were resolved through discussion or with the third reviewer (MS).

### Data synthesis

Studies were pooled in statistical meta-analysis using review manager (RevMan) software version 5.3 (42). Effect sizes were expressed as odds ratios and their 95% confidence intervals were calculated for analysis. Heterogeneity was assessed statistically using the standard *χ*^2^ and *I*^2^ tests. Statistical analysis was performed using the fixed effects model. Sensitivity analysis was conducted by excluding certain studies with relative effect change in a subgroup (36). It was also likely that the robustness of the review was checked against any changes in the analysis method. Where a presentation of all pooling data was not possible, the findings were presented in narrative form as appropriate.

### Assessing certainty in the findings

The Grading of Recommendations, Assessment, Development and Evaluation (GRADE) approach for grading the certainty of evidence was followed and a Summary of Findings (SoF) was created using GRADEPro GDT 2015 (McMaster University, ON, Canada) (43). The SoF was used to present the following information on main outcomes: incidence of complete expulsion or abortion with appropriate stratification, incomplete abortion, missed abortion, ongoing pregnancy for the treatment and control groups, estimates of relative risk, a ranking of the quality of the evidence based on the risk of bias, directness, heterogeneity, precision, and risk of publication bias of the review results (Table 2).

**Table 2:** Summary of findings.

Mifepristone plus misoprostol compared with misoprostol alone for first trimester medical abortion						
Outcomes	Anticipated absolute effects^a^ (95% CI)		Relative effect (95% CI)	No. of participants (studies)	Certainty of the evidence (GRADE)	Comments
	Risk with misoprostol alone	Risk with mifepristone plus misoprostol				
Complete expulsion based on gestational age follow-up: max. 63 days	775 per 1,000	922 per 1,000 (883 to 968)	RR 1.19 (1.14 to 1.25)	1,466 (5 RCTs)	⊕⊕⊕⊕ High	Although there is a moderate level of heterogeneity in the >49 days of the gestation subgroup, no significant difference was found between the treatment arms affecting the effect measure
Complete expulsion based on frequency/dosage of misoprostol (expulsion with dosage) assessed with ultrasound sonography and expert opinion	787 per 1,000	905 per 1,000 (873–944)	RR 1.15 (1.11–1.20)	2,052 (9 RCTs)	⊕⊕⊕○ Moderate^b^	Intervention group receiving either 400 mcg or 800 mcg of misoprostol after 48 h of mifepristone pre-treatment may not experience a high level of complete expulsion compared with the control group
Complete expulsion success with foetal heart beat (FHB)	787 per 1,000	905 per 1,000 (873–944)	RR 1.15 (1.11–1.20)	2,052 (9 RCTs)	⊕⊕⊕○ Moderate^c^	Despite a considerable level of overall and subgroup heterogeneity, the intervention may be more likely to induce complete expulsion compared with control
Complete expulsion success with the route of misoprostol administration	787 per 1,000	905 per 1,000 (873–944)	RR 1.15 (1.11–1.20)	2,052 (9 RCTs)	⊕⊕⊕○ Moderate^d^	Administration of misoprostol vaginally or buccally appears to more likely induce complete expulsion in the intervention group compared with the control group
Incomplete abortion	64 per 1,000	43 per 1,000 (29 to 61)	RR 0.67 (0.46–0.96)	2,052 (9 RCTs)	⊕⊕⊕○ Moderate^e^	The intervention may be more likely to reduce incomplete abortions compared with control
Missed abortion	56 per 1,000	12 per 1,000 (4–30)	RR 0.21 (0.08–0.54)	822 (2 RCTs)	⊕⊕⊕⊕ High	The intervention may significantly reduce the risks of missed abortions
Ongoing pregnancy	129 per 1,000	15 per 1,000 (6–34)	RR 0.12 (0.05–0.26)	822 (2 RCTs)	⊕⊕⊕⊕ High	The intervention may significantly reduce the risks of ongoing pregnancies

CI, confidence interval; RR, risk ratio.

GRADE Working Group grades of evidence.

High certainty: We are very confident that the true effect lies close to that of the estimate of the effect.

Moderate certainty: We are moderately confident in the effect estimate: the true effect is likely to be close to the estimate of the effect, but there is a possibility that it is substantially different.

Low certainty: Our confidence in the effect estimate is limited: the true effect may be substantially different from the estimate of the effect.

Very low certainty: We have very little confidence in the effect estimate: the true effect is likely to be substantially different from the estimate of the effect.

Explanations.

^a^ The risk in the intervention group (and its 95% confidence interval) is based on the assumed risk in the comparison group and the relative effect of the intervention (and its 95% CI).

^b^ There is a moderate level of heterogeneity among included studies in terms of effect by route of administration.

^c^ There is a high level of heterogeneity among included studies. Even a severe inconsistency is seen within each considered subgroup.

^d^ There is a moderate level of heterogeneity among included studies in terms of the route of misoprostol administration.

^e^ Pooled estimate shows a moderate level of heterogeneity between the considered subgroups and inconsistent report. In addition to a few studies, a significant level of heterogeneity exists in one subgroup.

## Results

### Study inclusion

A total of 1,10,594 studies were located using a systematic search of bibliographic databases and an additional 11 items were identified through a hand search. The databases searched were PubMed, EMBASE, and Cochrane CENTRAL. The hand search items comprised the WHO trial registry platform and Google Scholar. Of the 1,10,605 records, 13 duplicates were removed. A further screening of the titles and abstracts resulted in the exclusion of 10,559 items.

Two reviewers (TS and LB) independently reviewed the 33 full articles and excluded 23 articles as per the PICO criteria of the review question. Of these, one article was found as a conference abstract on a population of unknown gestational age, and a full text was not accessed since there was no reply from the author (44). A study by Dabash et al. (45) was excluded because the same study was published in an earlier volume of the same journal and contained the same co-authors who were included by us in the review (38). One article, which was published in 2012 and included in this review (37), was excluded since it was found to be republished by a different journal and had a sole author (46). The title by Ngo and Park (47) was a letter to the editor's commentary on a cautious interpretation of the conclusions in a study by Ngoc et al. (23). This study was included in our review.

**Figure 1:**
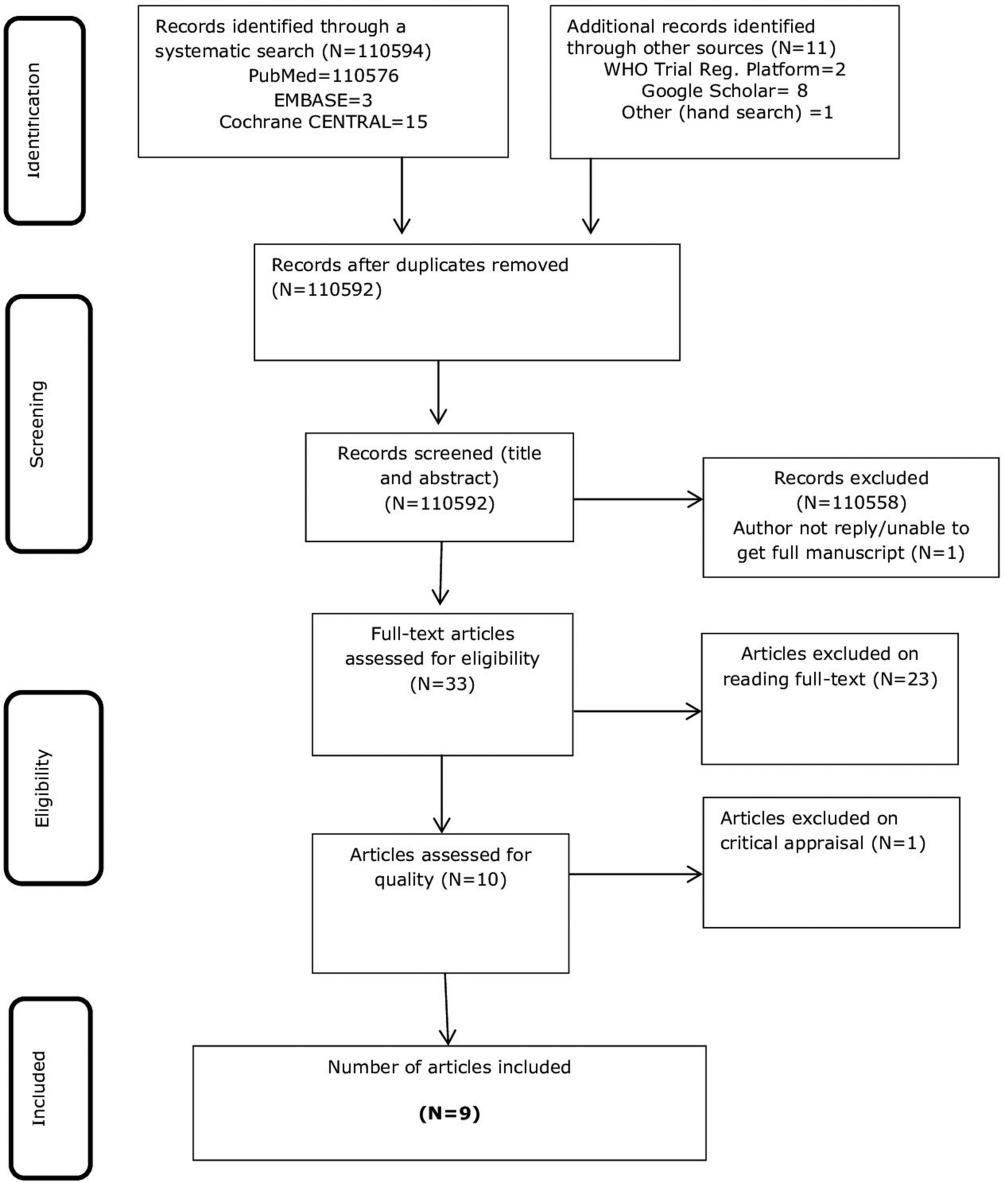
PRISMA flow chart of the study selection process (41).

Two conference abstracts of the same study were found to be published in different journals (48, 49). Accessing the full text of these studies was impossible as the authors did not reply to email requests. Nonetheless, a potential contribution of three excluded studies remains unknown unless the population is clearly defined in the first trimester (44) or a subgroup analysis has been presented for 9–12 weeks of gestation (48, 49). All the nine included studies (23, 30, 33–38, 50) were RCTs conducted in either teaching hospitals or maternity centres (Supplementary Appendix SIII). Two of the studies (30, 34) were conducted on populations with missed abortion or a blighted ovum. One study (51) conducted on a population of missed abortion was excluded on reasons of methodological quality. The study employed a crossover design with three treatment arms that included the groups of interest to this review, but a significant proportion of participants in the mifepristone plus misoprostol group had been intervened with surgical evacuation because of medication side effects. The fact that in five out of six participants in the mifepristone plus misoprostol group evacuation was done before commencing misoprostol might be a potential source of variation that will distort comparison. Details of the search and study selection process are shown in Figure 1. A list of all excluded studies is presented in the appendix (Supplementary Appendix SII) of the Supplementary File.

### Methodological quality

As presented in Table 1 (23, 30, 32, 34–38, 50), the methodological quality of the included studies was low for the following two questions: Q4: Were participants blind to treatment assignment? And Q5: Were those delivering treatment blind to treatment assignment? In four studies (30, 33, 34, 36), participants were not blinded to treatment assignment, while this was unclear in one study (37). In six of the studies (30, 33, 34, 36, 37, 50), healthcare personnel delivering treatment were not blinded. The quality of the included studies could be deemed adequate in the light of the 1st, 2nd, and last appraisal questions. Only three of the nine studies were characterized by the parameters of unclear (37) or no (33, 36) true randomization, allocation concealment, or appropriate design. The quality of the included studies could be regarded as high for the remaining evaluations (Table 1).

### Characteristics of included studies

#### Study design and setting

All the nine included studies (23, 30, 33–38, 50) were RCTs conducted in either teaching hospitals or maternity centres (Supplementary Appendix SIII). Two RCTs were conducted in the United States (30, 35), two in Tunisia (33, 50), one in Vietnam (23), one in Israel (34), one in India (37), one in Nepal (36), and one in Vietnam and Tunisia (38).

#### Participants

Data were extracted from a total of 2,052 pregnant women seeking induced medical abortion for either live or blighted ovum. Of these, 1,035 were assigned to the intervention (mifepristone followed by misoprostol) and 1,017 belonged to the control group assigned with the misoprostol-only regimen. The enrolled sample size of the studies ranged from 100 (36, 37) to 441 subjects (38) for both groups. The mean age of the participants ranged from 26 ± 6.0 years vs. 27 ± 6.0 years between the intervention and control groups, respectively, in the study by Jain et al. (35), to 32 ± 6.0 years (both groups) in the one by Stockheim et al. (34) Minimum and maximum age of women was reported by Blum et al. (38) to range from 15 to 45 (intervention group) to 18–46 (controls).

Three of the studies (35, 37, 50) reported the mean age of gestation in days ranging from 44.28 ± 5.93 (intervention) vs. 43.44 ± 6.54 (control) (37) to 47 ± 6.15 (intervention) vs. 47 ± 6.04 (control) groups (35). However, a higher mean gestational age of 10.1 ± 1.1 weeks was reported by Dalenda et al. (33).

There was no consistency in documenting gravidity and parity across the included studies. In the study by Ngoc et al. (23), the mean (SD) number of gravidity was small (2.5 ± 1.3 vs. 2.6 ± 1.5) compared with the highest figure reported by Fekih et al. (50) (3.35 ± 1.47 vs. 3.5 ± 1.9) between the intervention and the control groups, respectively. Meanwhile, the lowest and highest numbers of parity were reported in the study by Stockheim et al. (34), which ranged from 0 to 10 and a mean (SD) of 1.7 ± 1.8 vs. 1.4 ± 1.8 between the two respective groups.

In four studies that reported on marital status, Dalenda et al. (33) reported that about half of the participants were married, and in the rest of the studies (23, 38, 50), it was reported that more than two-third of the participants were married. Ethnic diversity among the participants was reported in the studies by Schreiber et al. (30) and Jain et al. (35).

#### Comparisons and treatment delivery

All of the included studies evaluated two arms of treatment: mifepristone plus misoprostol as an intervention and misoprostol only as a comparator regimen group. In four studies (23, 30, 37, 38), it was reported that the mifepristone pre-treatment group received 200 mg of oral mifepristone on day one, followed, 24 h later, by 800 mcg of misoprostol. In the study by Stockheim et al. (34), it was reported that 600 mg of the drug was used as part of pre-treatment. Of the five studies in which it was reported that misoprostol was administered after 48 h of giving mifepristone, two studies (33, 50) reported the use of a 400 mcg dose, whereas in three (34–36), patients reportedly received 800 mcg of the drug.

On the other hand, first-day misoprostol was delivered after placebo in control groups in three studies (23, 35, 38). In one of these studies (38), it was reported that a higher dose of 1,600 mcg was given 3 h apart on the second day, while 800 mcg dose was used buccally (23) and vaginally (35) on the third day of a placebo. In five other studies (30, 33, 36, 37, 50), it was reported that misoprostol was administered on the first day as a single dose of 800 mcg (4 tablets of 200 mcg), followed, 4 h, by another 800 mcg dose in one study (50). In their study, Stockheim et al. (34), however, reported that a 400 mcg oral dose was used 3 h apart. Four studies reported that the drug was used vaginally (30, 33, 35, 36), and four other studies showed that it was administered either sublingually (50) or buccally (23, 37, 38). The control groups in the study by Dahiya et al. (37) were administered with only a single dose of misoprostol on day one.

In both groups, either placebo or mifepristone was given to participants to swallow immediately, and additionally, misoprostol was provided to be taken at home. For the controls who were administered with misoprostol, either the provider had to insert the drug vaginally (30, 33, 36) or the participants were instructed to hold the drug buccally (37), take it sublingually (50), or consume it orally (34).

#### Outcomes

There was considerable similarity in the measurement of treatment outcomes across all included studies. The outcomes were classified as primary and secondary, as presented below. Subgroups were evaluated when appropriate.

Primary outcomes:

• Complete expulsion/abortion (23, 30, 33–38)
• Incomplete abortion (23, 30, 33–38, 50)
• Ongoing pregnancy (23, 38)
• Missed abortion (23, 38)

Secondary outcomes:

• Headache (30, 37)
• Fever (23, 30, 33–38, 50)
• Chills (23, 30, 33, 35–38)
• Nausea or vomiting (23, 30, 33, 35–38, 50)
• Diarrhoea (23, 30 33, 35–38, 50)
• Bleeding (subjective reporting) (23, 33, 38)
• Bleeding (mean hg count) (35, 36, 50)
• Pain (subjective reporting) (23, 30, 33, 35–38, 50)

#### Outcome assessment

In all cases, outcome was measured by using an expert's clinical evaluation and transvaginal sonography (ultrasound imaging). Participant appointments for outcome measurement ranged from the third day (48 h) of drug administration, in the study by Chawdhary et al. (36), through 1 week (23, 30, 37, 38) and 2 weeks (33, 34, 37, 50). In addition to post-intervention assessment, the participants were instructed to record, in their diary books, any side effects or events occurring at home. The details of included studies and population characteristics are summarized in Supplementary Appendix SIII.

### Review findings

#### Complete expulsion

**Figure 2:**
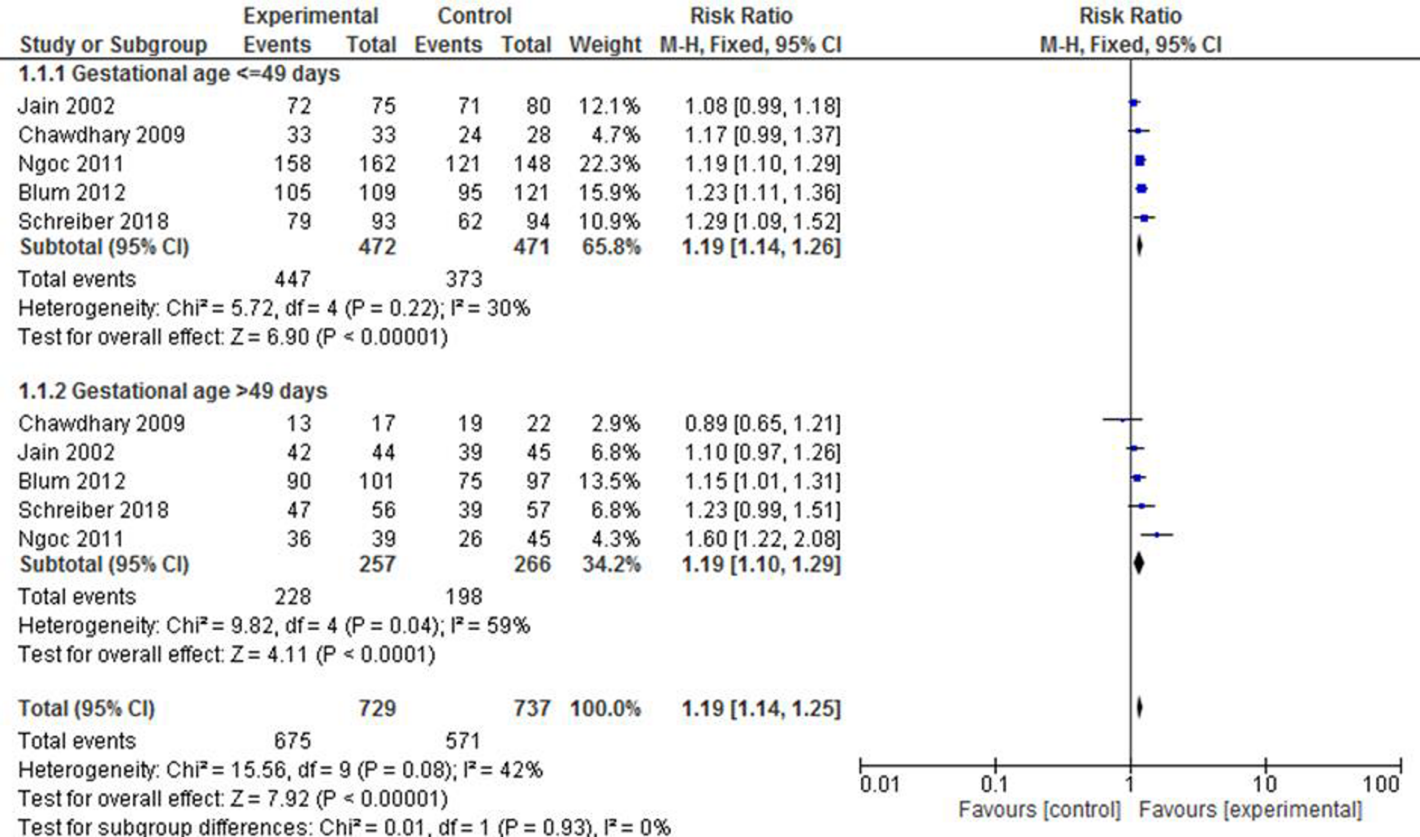
A gestational age-based subgroup meta-analysis of mifepristone plus misoprostol vs. misoprostol alone on complete abortion.

All included studies have reported findings on complete expulsion or abortion during the first trimester pregnancy. To minimize heterogeneity across the studies, a subgroup classification has been used on the basis of gestational age, frequency or dosage of misoprostol, and status of foetus heartbeat. Five studies (23, 30, 35, 36, 38) reported findings based on gestational age. The group with 49 or less days of gestation revealed that the experimental group is 19% times more likely to have complete expulsion compared with the control group (RR: 1.19; 95% CI: 1.14–1.26). It was likely that the subgroup with above 49 days of gestation showed a similar level of effectiveness between the two treatment groups (RR: 1.19; 95% CI: 1.10–1.29) despite the presence of a moderate level of heterogeneity (*I*^2 ^= 59%) (Figure 2).

**Figure 3:**
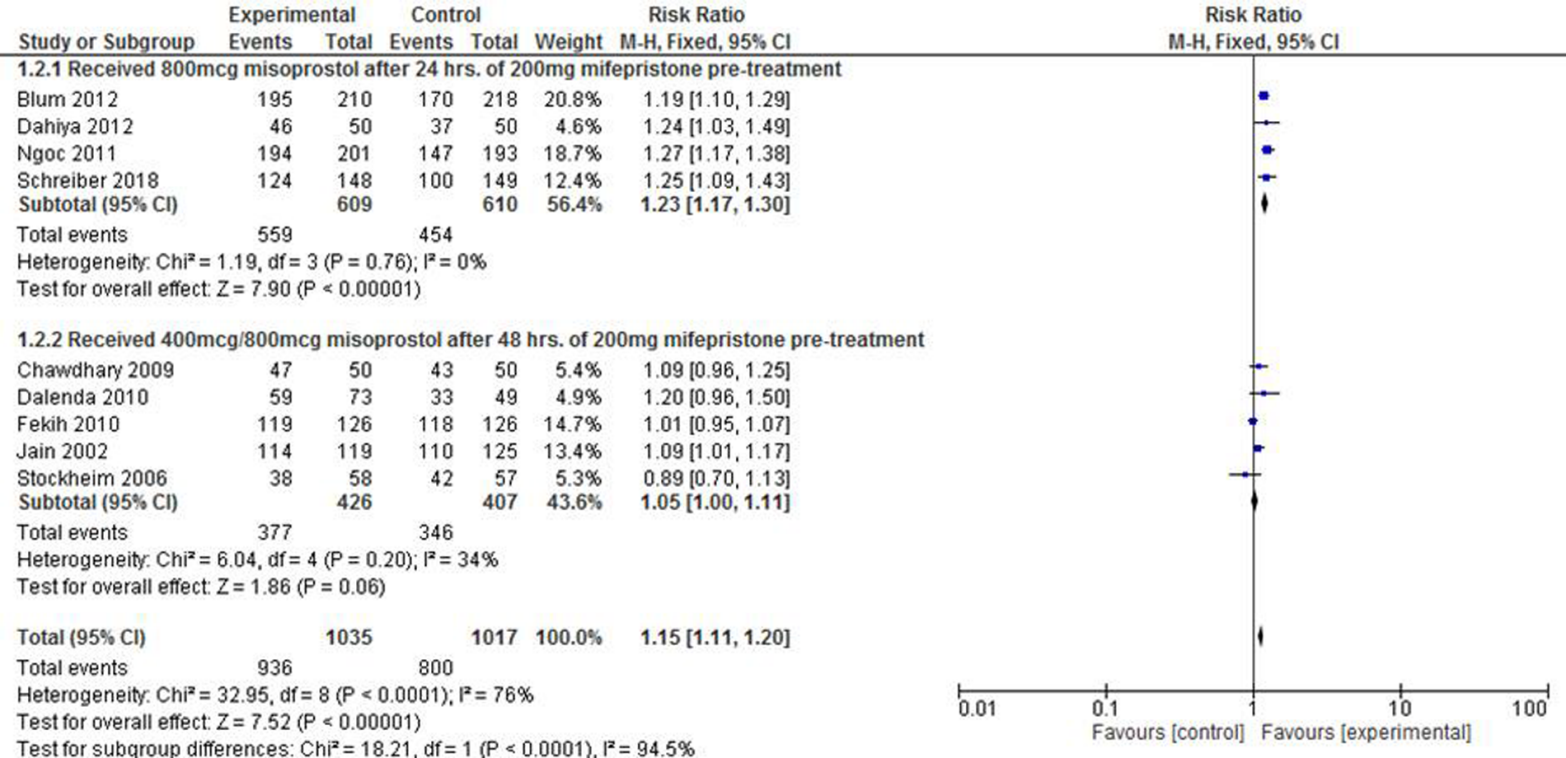
A misoprostol frequency/dosage-based subgroup meta-analysis of mifepristone plus misoprostol vs. misoprostol alone.

The potential variability in the success rate of complete expulsion due to the frequency or dosage of misoprostol was a factor of interest in this review. The pooled effect in four studies (23, 30, 37, 38) that documented the administration of 800 mcg misoprostol after 24 h of mifepristone pre-treatment showed that the intervention group was 1.23 times more likely to have complete expulsion compared with the control group (RR: 1.23; 95% CI: 1.17–1.30). In the remaining five studies (33–36, 50), however, it was found that the administration of either 400 mcg or 800 mcg of the drug after 48 h of mifepristone pre-treatment did not alter the success of complete expulsion between the groups compared (RR: 1.05; 95% CI: 1.00–1.11). The level of heterogeneity in both subgroups was also minimal (*I*^2 ^= 0%–34%) (Figure 3).

**Figure 4:**
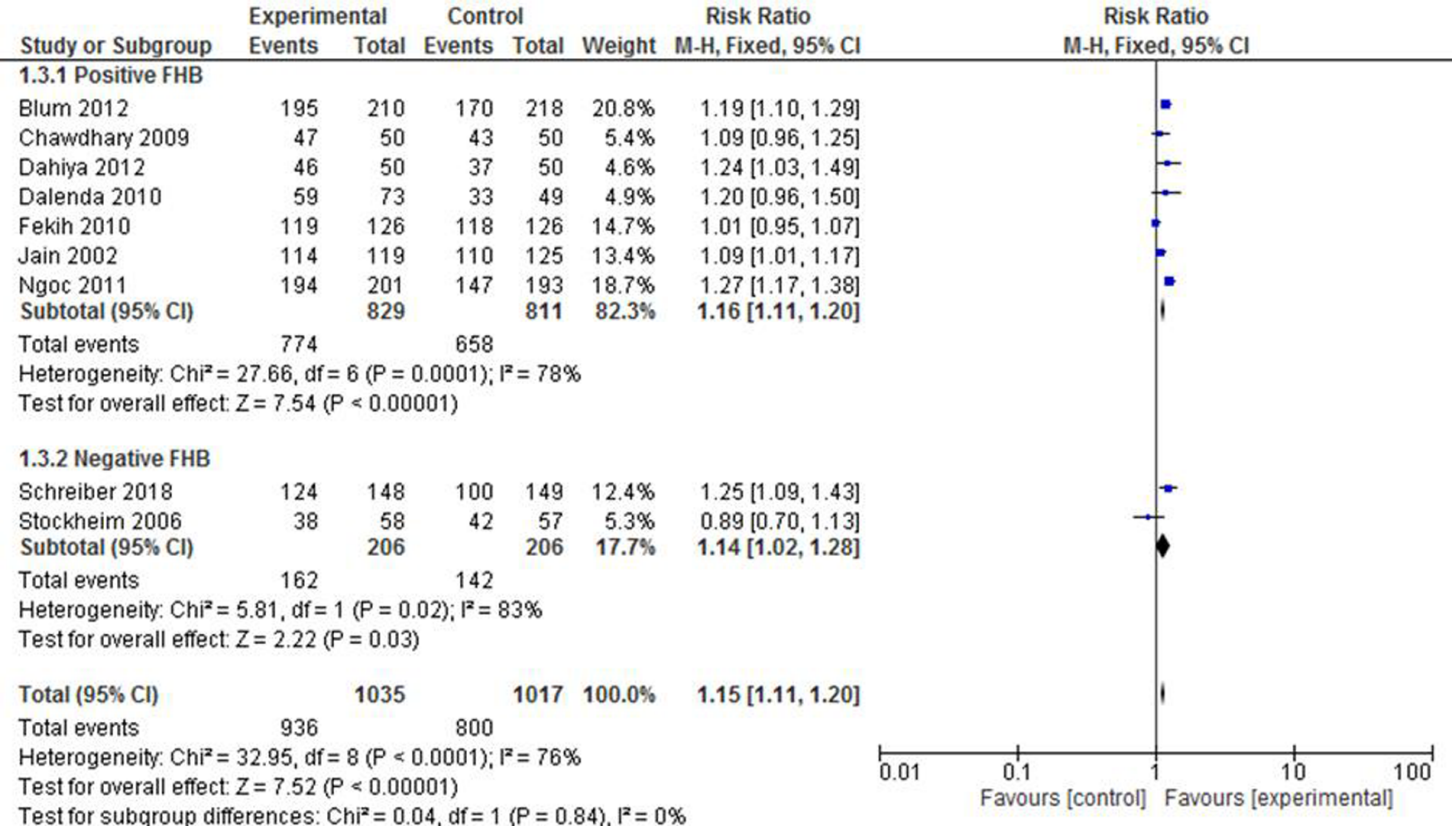
A FHB-based subgroup meta-analysis of mifepristone plus misoprostol vs. misoprostol alone.

The included studies were also stratified on the basis of foetal heartbeat (FHB). The pooled effect estimate of both subgroups showed a comparably similar likelihood of complete expulsion success between the intervention and the control groups (RR of 1.16 vs. 1.14). It is worth noting, however, that the included studies exhibited a considerable level of heterogeneity in both the positive and the negative FHB subgroups (*I*^2 ^= 78% and 83%, respectively) (Figure 4).

**Figure 5:**
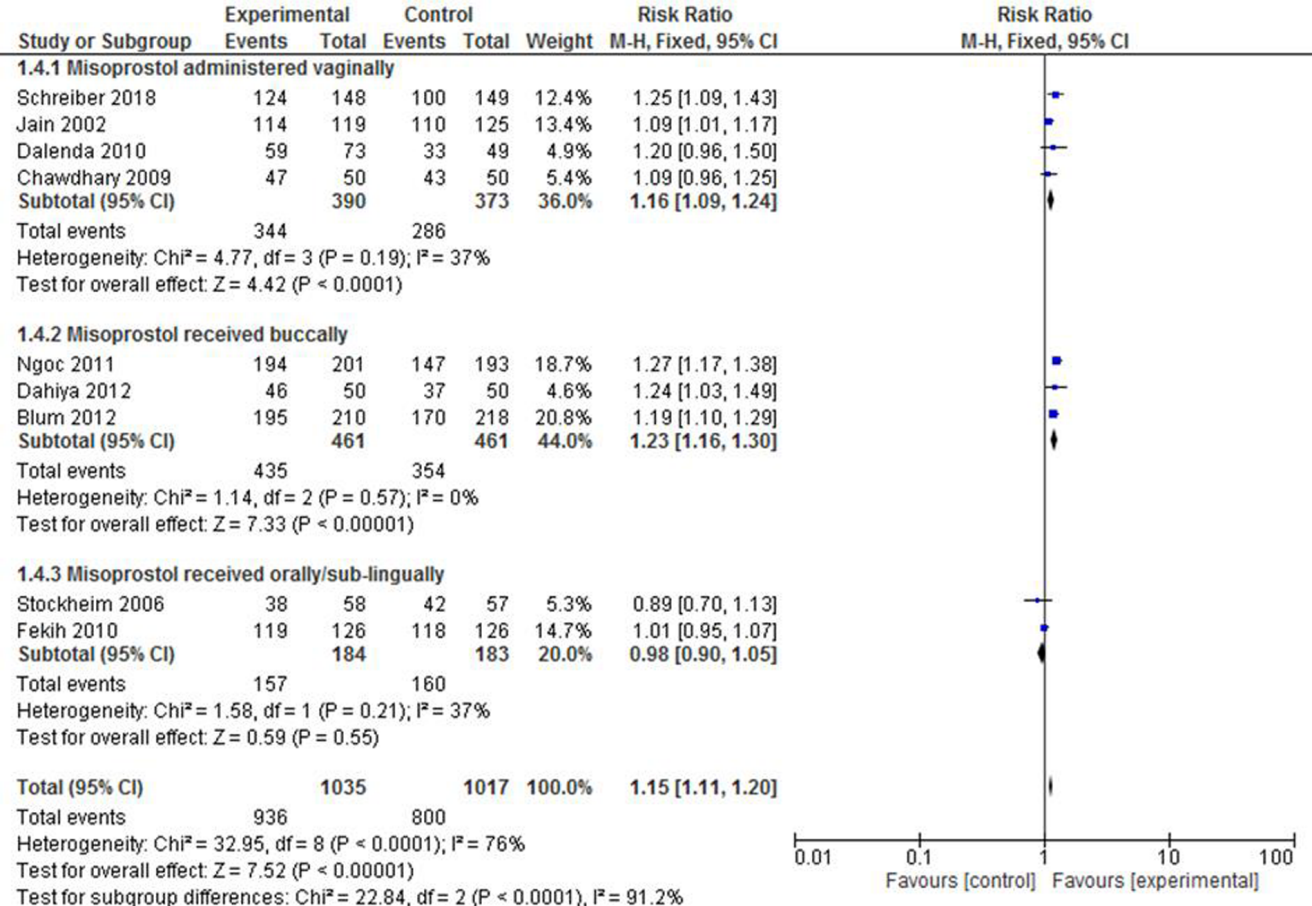
A route of administration-based subgroup meta-analysis of mifepristone plus misoprostol vs. misoprostol alone.

There was as statistically significant variation on the success rate of complete abortion among those in the subgroups who received misoprostol. The pooled risk ratio of four studies (30, 33, 35, 36) that reported the results of those who received the drug vaginally showed that the intervention group was 16% times more likely to experience complete expulsion than the control group (RR: 1.16; 95% CI: 1.09–1.24). The studies (23, 37, 38) that reported on the administration of the drug *via* the buccal route also revealed a superiority of the intervention group over the control group in terms of effect (RR: 1.23; 95% CI: 1.16–1.30. Meanwhile, there was no statistically significant difference between the two groups when misoprostol was administered orally or sublingually (34, 50). Although intra-sub-group heterogeneity was low across the groups (*I*^2 ^= 0%–37%), the test results for subgroup heterogeneity were statistically significant [*X*^2 ^= 22.84, df = 2, *p* < 0.0001; *I*^2^ = 91.2%] (Figure 5).

#### Incomplete abortion

**Figure 6:**
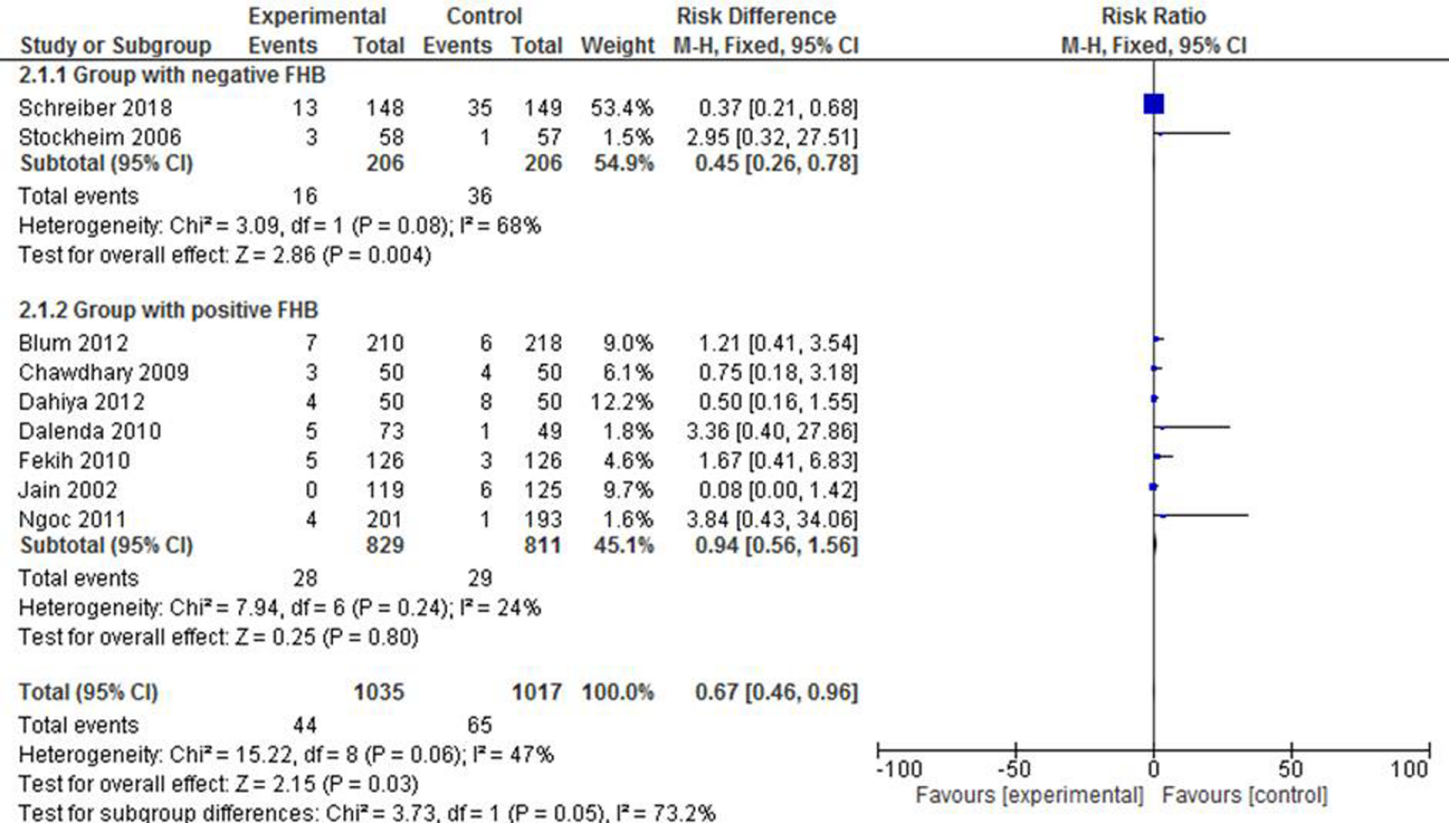
A FHB-based subgroup meta-analysis of mifepristone plus misoprostol vs. misoprostol alone on incomplete abortion.

Of all studies that evaluated the incidence of incomplete abortion, two studies (30, 34) reported a population with negative FHB, and it was treated as a separate group for analysis. Accordingly, it was shown that mifepristone plus misoprostol reduced the likelihood of incomplete abortion by 55% (RR: 0.45; 95% CI: 0.26–0.78) compared with the group that received misoprostol only. The heterogeneity level, here, was apparent [*X*^2 ^= 3.09, df = 1(*p* = 0.08); *I*^2 ^= 68%], as only two studies were included. The subgroup with positive FHB did not show any statistically significant difference between the intervention and the control groups (RR: 0.94; 95% CI: 0.56–1.56). The forest plot in Figure 6, however, shows that the RR of the overall effect estimation of incomplete abortion is reduced by 33% in the intervention group (RR: 0.67; 95% CI: 0.46–0.96) despite the consideration of a limited number of studies in one subgroup that might have led to the high level of overall heterogeneity [*X*^2 ^=^ ^15.22, df = 8 (*p* = 0.06); *I*^2 ^= 47%].

#### Missed abortion

**Figure 7:**
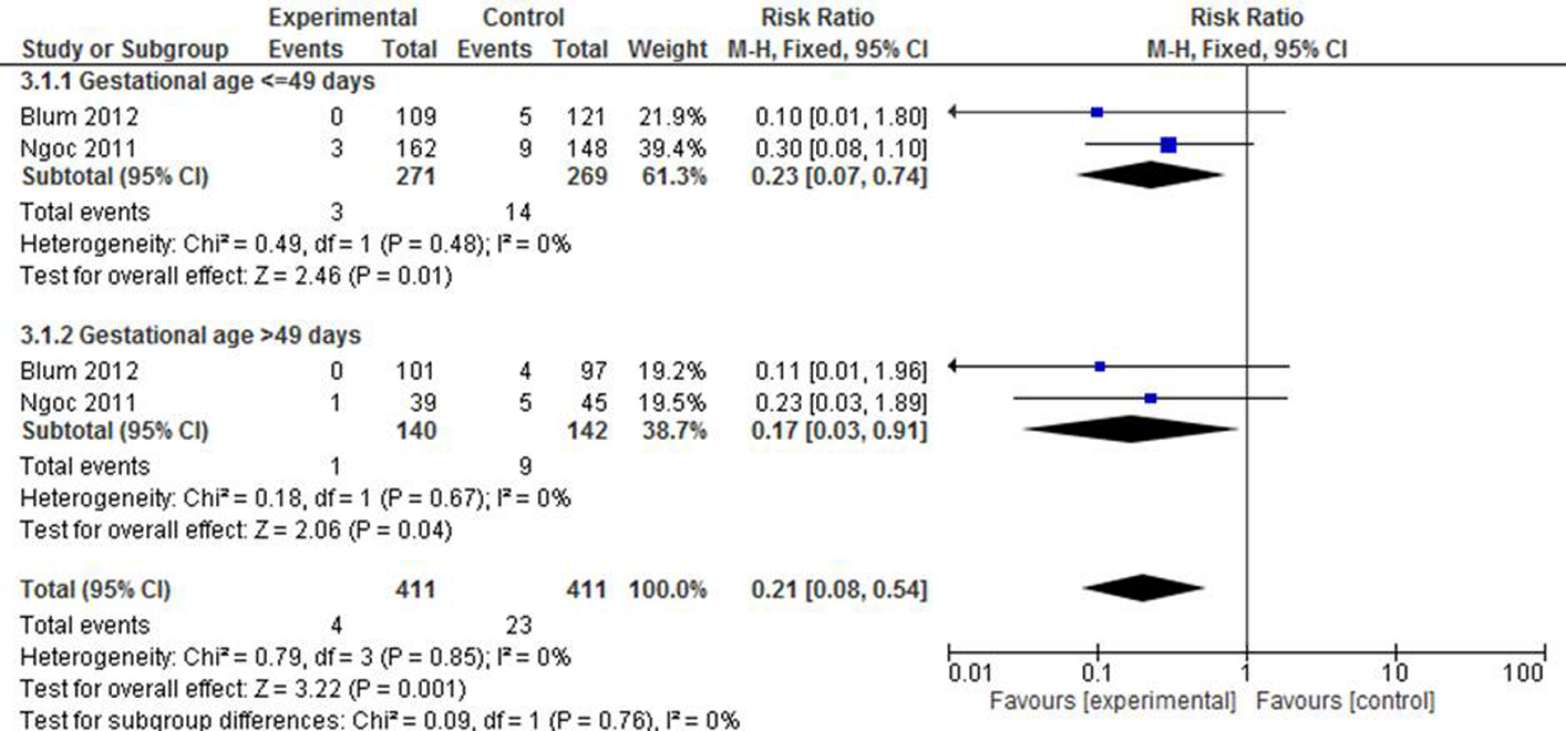
A gestational age-based subgroup meta-analysis of mifepristone plus misoprostol vs. misoprostol alone on missed abortion.

Figure 7 depicts a pooled effect measure of missed abortion on the basis of two studies (23, 38). A category of studies with a gestational age of either below or above 49 days demonstrated that there was a statistically significant prevention of missed abortion in the mifepristone plus misoprostol group (RR: 0.23; 95% CI: 0.07–0.74). Although the 95% confidence interval for both studies was higher in the second subgroup, there was no overall heterogeneity in the included studies [*X*^2 ^= 0.79, df = 1 (*p* = 0.85); *I*^2 ^= 0%].

#### Ongoing pregnancy

**Figure 8:**
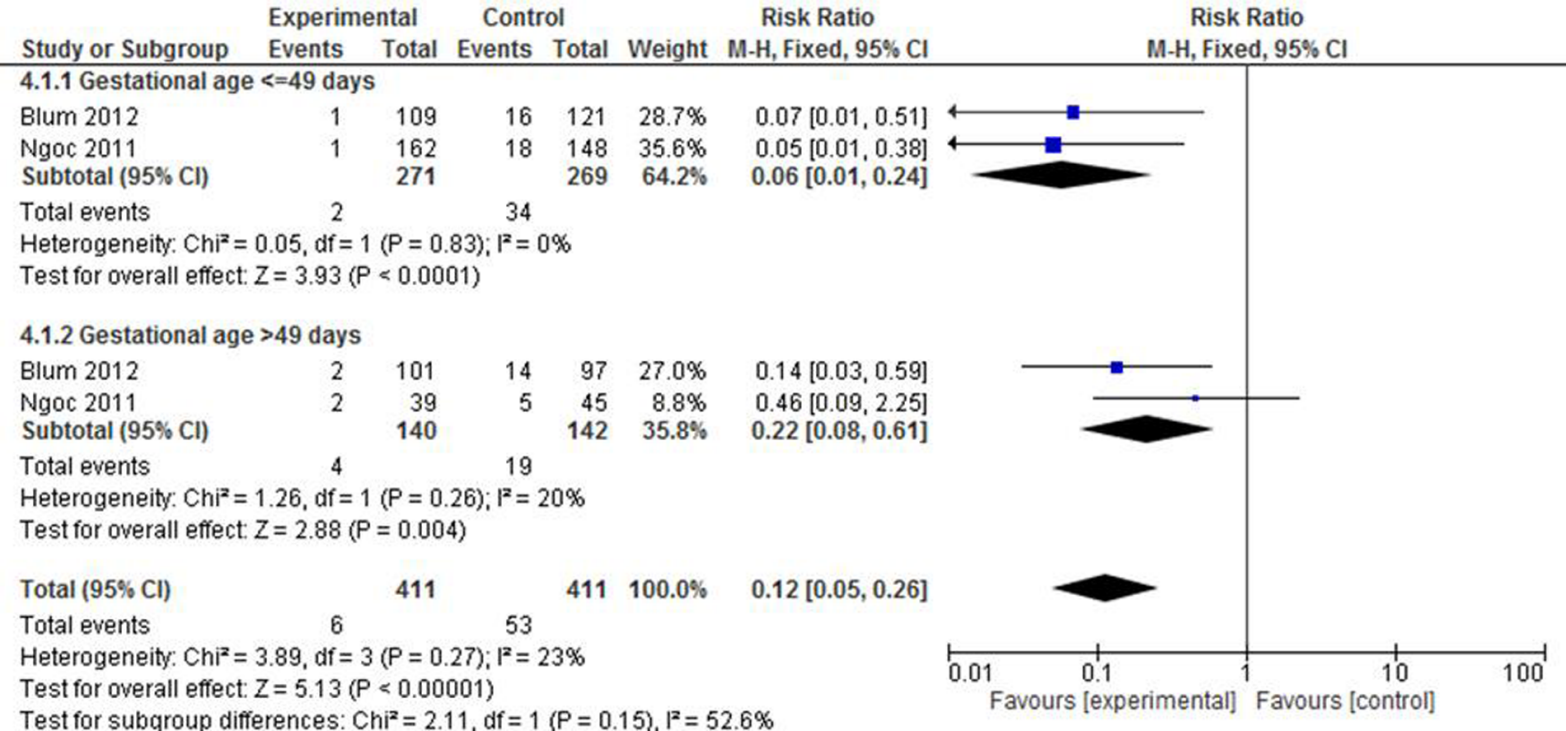
A gestational age-based subgroup meta-analysis of mifepristone plus misoprostol vs. misoprostol alone on ongoing pregnancy.

The studies by Ngoc et al. (23) and Blum et al. (38) reported on ongoing pregancy after the administration of the respective regimens. An evaluation of both below and above 49 days of gestation showed that mifepristone plus misoprostol significantly reduced the incidence of ongoing pregnacy (RR: 0.06; 95% CI: 0.01–0.24 and RR: 0.22; 95% CI: 0.08–0.61, respectively). The overall effect also shows that the intervention had resulted in a 88% contribution to reducing the risk of an ongoing pregnancy (RR: 0.12; 95% CI: 0.08–0.26) with a low level of heterogeneity among the included studies (Figure 8).

#### Secondary outcomes

The included studies also documented secondary outcomes measured either from patient diary records and reports for most of the cases as well as expert evaluation during treatment or at follow-up (35, 38, 50). One study (35) considered blood loss as a primary outcome.

Out of the seven studies that evaluated the presence of fever among 1,654 participants (835 intervention and 819 controls), only one study (50) showed a statistically significant protection from this side effect (RR: 0.33; 95% CI; 0.21–0.52). The overall pooled effect revealed a 22% decrease in side effects (RR: 0.78; 95% CI: 0.12–0.89) among the mifepristone plus misoprostol groups despite the presence of a high level of heterogeneity among the studies. Meanwhile, neither the individual nor the pooled effect measure of five studies (23, 30, 33, 37, 38) that reported on the chills or shivering status of 1,339 participants (680 intervention and 659 controls) showed evidence on the difference between the two groups (RR: 0.89; 95% CI: 0.77–1.04).

The presence of either nausea or vomiting or both was also reported in 1,935 participants in eight studies (975 intervention and 960 controls). However, there was no statistically significant difference between the two groups on the pooled effect size of this outcome (RR: 1.00; 95% CI: 0.94–1.06).Yet, there existed a considerable heterogeneity among the included studies [*X*^2 ^= 48.34, df = 7(*p* ≤ 0.001); *I*^2 ^= 86%]. It was likely that the pooled estimate did not show a significant difference on outcomes such as diarrhoea (RR: 0.90; 95% CI: 0.79–1.04), subjective report on pain (RR: 0.90; 95% CI: 0.79–1.02), and headache (RR: 1.19; 95% CI: 0.97–1.48). Only the overall estimate from four studies with 1,239 participants (630 intervention and 609 controls) showed that the subjective report of bleeding in the mifepristone plus misoprostol group was approximately 1.31 times higher than that in the control group (95% CI: 1.13–1.53). The included studies, however, were characterized by a high level of heterogeneity [*X*^2 ^= 17.61, df = 3(*p* ≤ 0.0005) *I*^2 ^= 83%].

In line with this, an objective measure on mean haemoglobin count was considered on the basis of reports from three studies (35, 36, 50). It was, indeed, not possible to pool their estimate since two of the studies (35, 50) did not report standard deviations from the mean. The studies reported that bleeding occurred for a duration of 7–14 days and was more frequent in the mifepristone plus misoprostol group, while a prolonged bleeding of over 14 days (that led to a significant fall in haematocrit count) was also noted in the group administered with the misoprostol-only regimen (36). In contrast, the report by Fekih et al. (50) documented that the mean haemoglobin decrease was in favour of misoprostol only (0.86 g/dl vs. 0.65 g/dl), while Jain et al. (35) reported that there was no significant difference between the groups.

## Discussion

Whereas earlier primary studies provided evidence of success for terminating early pregnancy applying either options (52, 53), reviews compared the clinical effectiveness of combined mifepristone and misoprostol against misoprostol alone for medical abortion (26, 27, 54). Although these reviews considered studies designed through RCTs, variations in the study population characteristics and outcomes evaluated make it difficult to draw linear conclusions across all settings. This review approached this question in a first trimester medical abortion and evaluated the incidence of four primary outcomes, namely, complete expulsion, incomplete abortion, missed abortion, and ongoing pregnancy, along with more secondary endpoints following the respective interventions.

Achieving a complete expulsion following any medical or surgical intervention of an unintended pregnancy is an ultimate goal where success is counted for clients and providers. While eight studies have reported on the expulsion rate following each option, an overall success rate in the experimental group was apparent in all cases. A subgroup analysis was performed on the basis of gestational age and foetal heartbeat to check for potential variations in effect strength. Accordingly, two (35, 36) out of the five studies with gestational age ≤49 days showed no strong evidence of variation in the odds of complete expulsion across the compared groups, whereas the rest revealed a superiority of the experimental group in terms of effect with a total average RR of 4.85 (95% CI: 3.04–7.75). In contrast, only two (23, 38) out of the five studies with gestational age >49 days showed a higher rate of achieving complete expulsion in the experimental group. Yet, the cumulative effect in this subgroup also showed that the combined mifepristone and misoprostol resulted in a 2.67 times higher likelihood of complete expulsion (95% CI: 1.66–4.29).

The fact that there exists a considerable level of heterogeneity in the studies counted could be one reason for the observed discrepancy. The small number of studies included in the evaluation could also contribute to the inconsistency. A recent systematic review (54) that evaluated two studies also documented a similar effect measured as successful abortion. The variation noted could be due to the fact that the earlier review did not report the finding in subgroups. By also evaluating individual studies, it could be speculated that either the dose of mifepristone or the route of misoprostol administration played a role in the outcome predicted. The dose of mifepristone in the study reported by Schreiber et al. (30) was only 200 mg, whereas Jain et al. (35) and Chawdhary et al. (36) administered a placebo instead, and all these three considered the vaginal route of misoprostol. Compared with others, these studies revealed an effect of no difference despite adjusting for gestational age. While there exists evidence on poor absorption of vaginally administered misoprostol (55), a retrospective review has also documented that reduced doses of mifepristone and vaginally administered misoprostol produced complete abortion in up to 95% of cases (56). This too is in agreement with another systematic report that documented that failure of medical abortion was associated with the oral route of misoprostol and increased gestational age (57). In line with this, a study in China showed that a 400 mcg sublingual misoprostol and 800 mcg misoprostol administered vaginally resulted in an invariably successful and complete abortion, which however, earlier revealed a shorter time to the outcome and reduced adverse events, suggesting the option of devising an optimal strategy (58). Adjusting for foetal heart beat (FHB), one (34) out of two studies that reported negative FHB and three (33, 36, 50) out of seven that documented positive FHB showed no significant difference. The overall effect of complete abortion in the positive FHB group was 2-fold (RR: 1.65 vs. 3.54), but with a significant heterogeneity and small sample size in the negative FHB group. By assessing for variation within the trial group, a study revealed that a high level of efficacy in the termination of 10–16 weeks’ pregnancy was observed in groups receiving the compound regimen (35 mg of mifepristone qd, followed, 12 h after the second dose, by 0.6 mg of vaginal misoprostol soaked in 0.9% humidified NaCl) compared with the group receiving the combined regimen (50 mg of mifepristone bid, followed 12 h after the third administration). The study also reported no difference in missed abortion treatment (59). On the other hand, the combined regimen resulted in an overall reduction in the rate of incomplete abortions by 35%, whereas it remained inconclusive among the subgroups with positive FHB. The fact that only two studies (30, 34), exhibiting a substantial heterogeneity, reported negative FHB might make it difficult to conclude that the observed success in the reduction of the same outcome was higher in the experimental group. It was likely that missed abortion substantially reduced by 79% in the experimental group, and no profound variation was noted by gestational age. This was also in agreement with a multicentre-based RCT report in the United Kingdom (60). In the same fashion, two studies (23, 38) that evaluated the effect of the combined regimen against ongoing pregnancy revealed a high level of effectiveness. This was also documented in other systematic reviews (32, 54) that reported on a population of varied gestational age.

Our extended aim in this review was to evaluate the incidence of adverse outcomes in both treatment arms. No statistically significant difference was noted in terms of nausea and/or vomiting, diarrhoea, pain, and headache across the two groups. The pooled estimate for fever and chills was lower in the experimental group by 36% and 21%, respectively, whereas the subjective report of bleeding showed a 62% higher likelihood in the same group. While other studies suggest that these side effects are common in both groups (61, 62), the observed discrepancy may be attributed to the heterogeneity of the included studies. Also, available evidence states that either the combined regimen reduces blood loss (25) or that there is no significant variation between groups (61). However, the disparity in the higher odds of the subjective report of blood loss, in this review, could be due to the consideration of few studies. The subjective experiences of participants may not substitute for an objective outcome, which, in turn, makes it difficult to draw a precise conclusion. Given all these, however, it is important to note that the safety and success of outcomes from both regimens may heavily depend on other factors such as providers' skill, settings, and adequacy of information, as highlighted by Ganatra et al. (63), which, in turn, makes misoprostol alone a safe method in settings where the combined regimen is not accessible. There is an effort in this review to evaluate more endpoints to pool reports on the comprehensive effects of the respective regimens. It also has attempted to consider a subgroup analysis by gestational age, FHB, route of administration, and frequency as well as the varied strengths of misoprostol. However, the reviewers would also like to admit the limitation that only RCTs have been included and evaluated, which potentially could undermine the incidence of rare or long-term adverse outcomes following each treatment. In addition, the inclusion of articles published only in the English language and the reviewers' incapability to access some subscription-only journals might have introduced an element of bias in this review.

## Conclusion

This review indicated and further strengthened the fact that the combined mifepristone and misoprostol regimen could be an effective medical management strategy for inducing abortions during first trimester pregnancy in all contexts. Specifically, there is a high-level certainty of evidence on complete expulsion during the early stage and its ability to reduce both missed and ongoing pregnancies. The reviewers recommend for inclusion and evaluations of observational studies that assess the long-term adverse outcomes of each treatment strategy. The effectiveness of the intervention in the late gestational age of pregnancy should also be examined to guide current practice.

### Registration and protocol

This systematic review and meta-analysis has been registered on the International Register of Systematic Review Protocols (PROSPERO) (No. CRD42019134213) (64). Further, a review protocol has been developed and was published (65). No ethical approval was sought because this was a systematic review and meta-analysis of published literature.

## Data Availability

The original contributions presented in the study are included in the article/**Supplementary Material;** further inquiries can be directed to the corresponding author.

## References

[1] Costescu D Guilbert E Bernardin J Black A Dunn S Fitzsimmons B Medical abortion. J Obstet Gynaecol Can. (2016) 38(4):366–89. 10.1016/j.jogc.2016.01.002 27208607

[2] Upadhyay UD Desai S Zlidar V Weitz TA Grossman D Anderson P Incidence of emergency department visits and complications after abortion. Obstet Gynecol. (2015) 125(1):175–83. 10.1097/AOG.0000000000000603 25560122

[3] Xu W Zhang W Zhang X Dong T Zeng H Fan Q. Association between formaldehyde exposure and miscarriage in Chinese women. BioMed Res Int. (2017) 96(26):e7146. 10.1097/MD.0000000000007146 PMC5500027 28658105

[4] Andersen JT Mastrogiannis D Andersen NL Petersen M Broedbaek K Cejvanovic V Diclofenac/misoprostol during early pregnancy and the risk of miscarriage: a Danish nationwide cohort study. Arch Gynecol Obstet. (2016) 294(2):245–50. 10.1007/s00404-015-3966-9 26585175

[5] Cederbaum JA Putnam-Hornstein E Sullivan K Winetrobe H Bird M. STD and abortion prevalence in adolescent mothers with histories of childhood protection involvement. Perspect Sex Reprod Health. (2015) 47(4):187–93. 10.1363/47e4215 26148780

[6] Jones HE O’Connell White K Norman WV Guilbert E Lichtenberg ES Paul M. First trimester medication abortion practice in the United States and Canada. PloS One. (2017) 12(10):e0186487. 10.1371/journal.pone.0186487 29023594 PMC5638562

[7] Purcell C Cameron S Lawton J Glasier A Harden J. Self-management of first trimester medical termination of pregnancy: a qualitative study of women’s experiences. BJOG. (2017) 124(13):2001–8. 10.1111/1471-0528.14690 28421651 PMC5724679

[8] van den Berg J Gordon BB Snijders MP Vandenbussche FP Coppus SF. The added value of mifepristone to non-surgical treatment regimens for uterine evacuation in case of early pregnancy failure: a systematic review of the literature. Eur J Obstet Gynecol Reprod Biol. (2015) 195:18–26. 10.1016/j.ejogrb.2015.09.027 26461963

[9] Louie KS Chong E Tsereteli T Avagyan G Vardanyan S Winikoff B. The introduction of first trimester medical abortion in Armenia. Reprod Health Matters. (2015) 22(44 Suppl 1):56–66. 10.1016/S0968-8080(15)43824-8 25702069

[10] Lo SS Ho PC. First-trimester medical abortion service in Hong Kong. Hong Kong Med J. (2015) 21(5):462–7. 10.12809/hkmj154525 26493078

[11] Jones RK Nash E Cross L Philbin L Kirstein M. Medication abortion now accounts for more than half of all us abortions (2022). Available at: https://www.guttmacher.org/article/2022/02/medication-abortion-now-accounts-more-half-all-us-abortions (Accessed February 5, 2023).

[12] Kortsmit K Nguyen AT Mandel MG Clark E Hollier LM Rodenhizer J Abortion surveillance—United States, 2020. MMWR Surveill Summ. (2022) 71(SS-10):1–27. 10.15585/mmwr.ss7110a 36417304 PMC9707346

[13] Bygdeman M Danielsson KG. Options for early therapeutic abortion: a comparative review. Drugs. (2002) 62(17):2459–70. 10.2165/00003495-200262170-00005 12421103

[14] Chen C Lin F Wang X Jiang Y Wu S. Mifepristone combined with ethacridine lactate for the second-trimester pregnancy termination in women with placenta previa and/or prior cesarean deliveries. Arch Gynecol Obstet. (2017) 295(1):119–24. 10.1007/s00404-016-4205-8 27658386

[15] Li CL Song LP Tang SY Zhou L He H Mo XT Efficacy, safety, and acceptability of low-dose mifepristone and self-administered misoprostol for ultra-early medical abortion: a randomized controlled trial. Reprod Sci. (2017) 24(5):731–7. 10.1177/1933719116669055 27678099

[16] Goldstone P Walker C Hawtin K. Efficacy and safety of mifepristone-buccal misoprostol for early medical abortion in an Australian clinical setting. Aust N Z J Obstet Gynaecol. (2017) 57(3):366–71. 10.1111/ajo.12608 28303569

[17] Shrestha D Aryal S Safety SB. Efficacy and acceptability of early first trimester abortion using oral mifepristone and sublingual misoprostol. J Nepal Health Res Counc. (2018) 16(3):269–73. 10.33314/jnhrc.v16i3.1378 30455484

[18] Kulier R Gülmezoglu AM Hofmeyr GJ Cheng LN Campana A. Medical methods for first trimester abortion. Cochrane Database Syst Rev. (2004) 1:CD002855. 10.1002/14651858.CD002855.pub4 14973995

[19] Calder A. The clinical use of prostaglandins for early and late abortion. In: Hillier K, editors. Eicosanoids and reproduction. Advance in eicosanoid research. Vol 1. Dordrecht: Springer (1987). pp. 184–94. 10.1007/978-94-009-3215-9_9

[20] Alfirevic Z Keeney E Dowswell T Welton NJ Dias S Jones LV Labour induction with prostaglandins: a systematic review and network meta-analysis. Br Med J. (2015) 350:h217. 10.1136/bmj.h217 25656228 PMC4353287

[21] Mahajan DK London SN. Mifepristone (RU486): a review. Fertil Steril. (1997) 68(6):967–76. 10.1016/S0015-0282(97)00189-1 9418681

[22] Schaff EA Eisinger SH Stadalius LS Franks P Gore BZ Poppema S. Low-dose mifepristone 200mg and vaginal misoprostol for abortion. Contraception. (1999) 59(1):1–6. 10.1016/S0010-7824(98)00150-4 10342079

[23] Ngoc NTN Shochet T Raghavan S Blum J Nga NTB Minh NTH Mifepristone and misoprostol compared with misoprostol alone for second-trimester abortion: a randomized controlled trial. Obstet Gynecol. (2011) 118(3):601–8. 10.1097/AOG.0b013e318227214e 21860289

[24] Patel U Chauhan K Singhi S Kanani M. Second trimester abortion-mifepristone and misoprostol or misoprostol alone? Int J Reprod Contracept Obstet Gynecol. (2013) 2(3):315–9. 10.5455/2320-1770.ijrcog20130911

[25] Akkenapally PL. A comparative study of misoprostol only and mifepristone plus misoprostol in second trimester termination of pregnancy. J Obstet Gynaecol India. (2016) 66(Suppl 1):251–7. 10.1007/s13224-016-0869-z 27651613 PMC5016460

[26] Gemzell-Danielsson K Lalitkumar S. Second trimester medical abortion with mifepristone–misoprostol and misoprostol alone: a review of methods and management. Reprod Health Matters. (2008) 16(Suppl 31):162–72. 10.1016/S0968-8080(08)31371-8 18772097

[27] Hammond C. Recent advances in second-trimester abortion: an evidence-based review. Am J Obstet. Gynecol. (2009) 200(4):347–56. 10.1016/j.ajog.2008.11.016 19318143

[28] Hapangama D Neilson JP. Mifepristone for induction of labour. Cochrane Database Syst Rev. (2009) 8(3):CD002865. 10.1002/14651858.CD002865.pub2 PMC3992376 19588336

[29] Singh S Remez L Sedgh G Kwok L Onda T. Abortion worldwide 2017: Uneven progress and unequal access. New York: Guttmacher Institute (2018). 1–68. Available at: https://www.guttmacher.org/sites/default/files/report_pdf/abortion-worldwide-2017.pdf (Accessed January 20, 2023).

[30] Schreiber CA Creinin MD Atrio J Sonalkar S Ratcliffe SJ Barnhart KT. Mifepristone pretreatment for the medical management of early pregnancy loss. N Engl J Med. (2018) 378(23):2161–70. 10.1056/NEJMoa1715726 29874535 PMC6437668

[31] Chen MJ Creinin MD. Mifepristone with buccal misoprostol for medical abortion: a systematic review. Obstet Gynecol. (2015) 126(1):12–21. 10.1097/AOG.0000000000000897 26241251

[32] Kapp N Baldwin MK Rodriguez MI. Efficacy of medical abortion prior to 6 gestational weeks: a systematic review. Contraception. (2018) 97(2):90–9. 10.1016/j.contraception.2017.09.006 28935220

[33] Dalenda C Ines N Fathia B Malika A Bechir Z Ezzeddine S Two medical abortion regimens for late first-trimester termination of pregnancy: a prospective randomized trial. Contraception. (2010) 81(4):323-7. 10.1016/j.contraception.2009.12.002 20227549

[34] Stockheim D Machtinger R Wiser A Dulitzky M Soriano D Goldenberg M A randomized prospective study of misoprostol or mifepristone followed by misoprostol when needed for the treatment of women with early pregnancy failure. Fertil Steril. (2006) 86(4):956–60. 10.1016/j.fertnstert.2006.03.032 17027362

[35] Jain JK Dutton C Harwood B Meckstroth KR Mishell DR. A prospective randomized, double-blinded, placebo-controlled trial comparing mifepristone and vaginal misoprostol to vaginal misoprostol alone for elective termination of early pregnancy. Hum Reprod. (2002) 17(6):1477–82. 10.1093/humrep/17.6.1477 12042265

[36] Chawdhary R Rana A Pradhan N. Mifepristone plus vaginal misoprostol vs vaginal misoprostol alone for medical abortion in gestation 63 days or less in Nepalese women: a quasi-randomized controlled trial. J Obstet Gynaecol Res. (2009) 35(1):78–85. 10.1111/j.1447-0756.2008.00864.x 19215552

[37] Dahiya K Ahuja K Dhingra A Duhan N Nanda S. Efficacy and safety of mifepristone and buccal misoprostol versus buccal misoprostol alone for medical abortion. Arch Gynecol Obstet. (2012) 285(4):1055–8. 10.1007/s00404-011-2110-8 22009509

[38] Blum J Raghavan S Dabash R Ngoc NTN Chelli H Hajri S Comparison of misoprostol-only and combined mifepristone-misoprostol regimens for home-based early medical abortion in Tunisia and Vietnam. Int J Gynaecol Obstet. (2012) 118(2):166–71. 10.1016/j.ijgo.2012.03.039 22682768

[39] Tufanaru C Munn Z Aromataris E Campbell J Hopp L. Chapter 3: systematic reviews of effectiveness. In: Aromataris E Munn Z, editors. JBI manual for evidence synthesis. Adelaide: JBI (2020). pp 71–132. Available at: https://synthesismanual.jbi.global (Accessed January 20, 2023). 10.46658/JBIMES-20-04

[40] Munn Z Aromataris E Tufanaru C Stern C Porritt K Farrow J The development of software to support multiple systematic review types: the Joanna Briggs Institute System for the Unified Management, Assessment and Review of Information (JBI SUMARI). Int J Evid Based Healthc. (2019) 17(1):36–43. 10.1097/XEB.0000000000000152 30239357

[41] Moher D Liberati A Tetzlaff J Altman DG; The PRISMA Group. Preferred reporting items for systematic reviews and meta-analyses: the PRISMA statement. PLoS Med. (2009) 6(7):e1000097. 10.1371/journal.pmed.1000097 19621072 PMC2707599

[42] The Nordic Cochrane Centre, The Cochrane Collaboration. Review manager (RevMan). version 5.3. Copenhagen: The Nordic Cochrane Centre, The Cochrane Collaboration (2014). Version 5.3. Available at: https://training.cochrane.org/online-learning/core-software/revman (Accessed January 20, 2023).

[43] GRADEpro. GDT: GRADEpro guideline development tool [Software]. McMaster University, (Developed by Evidence Prime, Inc.) (2015). Available at: gradepro.org. (Accessed August 2022).

[44] Bracken H Zuberi N de Guevara Puerto AL Mayi-Tsonga S Buendía Gómez M Irfan Ahmed S Mifepristone and sublingual misoprostol versus sublingual misoprostol alone for missed abortion: results of a randomized placebo-controlled trial. Contraception. (2019) 99(5):315–6. 10.1016/j.contraception.2019.03.005

[45] Dabash R Blum J Raghavan S Ngoc NTN Chelli H Hajri S Outcomes of a double-blind randomized trial comparing misoprostol-only to mifepristone+misoprostol for home-based early medical abortion. Int J Gynaecol Obstet. (2012) 119:S315. 10.1016/S0020-7292(12)60585-2 22682768

[46] Dahiya K. Randomized trial of mifepristone and buccal misoprostol vs misoprostol alone for medical abortion. Int J Gynaecol Obstet. (2015) 131:E589. 10.1007/s00404-011-2110-8

[47] Ngo TD Park MH. Mifepristone+misoprostol vs. misoprostol alone for early medical abortion. Contraception. (2012) 85(2):219; author reply 219–20. 10.1016/j.contraception.2011.06.005 22067779

[48] Ngoc N Blum J Nga N Raghavan S Winikoff B. Medical abortion with misoprostol only versus mifepristone plus misoprostol: results from a randomized controlled trial. Int J Gynaecol Obstet. (2009) 107:S286.

[49] Blum J Ngoc NT Nga NT Raghavan S Winikoff B. Medical abortion with misoprostol only vs. mifepristone plus misoprostol: results from a randomized controlled trial. Contraception. (2009) 80(2):P195. 10.1016/j.contraception.2009.05.005

[50] Fekih M Fathallah K Ben Regaya L Bouguizane S Chaieb A Bibi M Sublingual misoprostol for first trimester termination of pregnancy. Int J Gynaecol Obstet. (2010) 109(1):67–70. 10.1016/j.ijgo.2009.11.008 20053398

[51] Grønlund A Grønlund L Clevin L Andersen B Palmgren N Lidegaard Ø. Management of missed abortion: comparison of medical treatment with either mifepristone+misoprostol or misoprostol alone with surgical evacuation. A multi-center trial in Copenhagen county, Denmark. Acta Obstet Gynecol Scand. (2002) 81(11):1060–5. PMID: 12421175

[52] Zikopoulos KA Papanikolaou EG Kalantaridou SN Tsanadis GD Plachouras NI Dalkalitsis NA Early pregnancy termination with vaginal misoprostol before and after 42 days gestation. Hum Reprod. (2002) 17(12):3079–83. 10.1093/humrep/17.12.3079 12456606

[53] Spitz IM Bardin CW Benton L Robbins A. Early pregnancy termination with mifepristone and misoprostol in the United States. N Engl J Med. (1998) 338(18):1241–7. 10.1056/NEJM199804303381801 9562577

[54] Abubeker FA Lavelanet A Rodriguez MI Kim C. Medical termination for pregnancy in early first trimester (≤63 days) using combination of mifepristone and misoprostol or misoprostol alone: a systematic review. BMC Womens Health. (2020) 20(1):142. 10.1186/s12905-020-01003-8 32635921 PMC7339463

[55] World Health Organization, Department of Reproductive Health and Research. Safe abortion: technical and policy guidance for health systems. 2nd ed. Geneva: WHO (2012). Available at: http://apps.who.int/iris/bitstream/10665/70914/1/9789241548434_eng.pdf?ua=1 (Accessed June 15, 2021). 23700650

[56] Anjum ZK. Termination of early pregnancy with a reduced oral dose of mifepristone and vaginal misoprostol. S Afr Med J. (2000) 90(9):889–91. PMID: 11081141

[57] Raymond EG Shannon C Weaver MA Winikoff B. First-trimester medical abortion with mifepristone 200mg and misoprostol: a systematic review. Contraception. (2013) 87(1):26–37. 10.1016/j.contraception.2012.06.011 22898359

[58] Libei D Wun Raymond RH Waixiang Y Yuqi Y Tingting R Chung HP. Comparison of the efficacy of 400 μg sublingual misoprostol versus 800 μg vaginal misoprostol for medical abortion in early pregnancy. Chin J Clinical Rational Drug Use. (2022) 15(11):23–7. 10.15887/j.cnki.13-1389/r.2022.11.007

[59] Bei-ying A Xiao-cui L Hai-yun W. Clinical trial of compound mifepristone tablets combined with misoprostol tablets in the treatment of termination pregnancy from 10 to 16 weeks and missed abortion. Chin J Clin Pharmacol. (2017) 33(6):499–503. 10.13699j.cnki.1001-6821.2017.06.006

[60] Chu JJ Devall AJ Beeson LE Hardy P Cheed V Sun Y Mifepristone and misoprostol versus misoprostol alone for the management of missed miscarriage (MifeMiso): a randomised, double-blind, placebo-controlled trial. Lancet. (2020) 396(10253):770–8. 10.1016/S0140-6736(20)31788-8 32853559 PMC7493715

[61] Stanulov G Anthoulaki X Deuteraiou D Chalkidou A Tsikouras P Path W Comparative study for efficacy of termination in first trimester pregnancy using misoprostol and mifepristone. Arch Community Med Public Health. (2018) 4(2):38–46. 10.17352/2455-5479.000037

[62] Nagaria T Sirmor N. Misoprostol vs mifepristone and misoprostol in second trimester termination of pregnancy. J Obstet Gynaecol India. (2011) 61(6):659–62. 10.1007/s13224-011-0118-4 23204686 PMC3307930

[63] Ganatra B Gerdts C Rossier C Johnson BR Tunçalp Ö Assifi A Global, regional, and subregional classification of abortions by safety, 2010–14: estimates from a Bayesian hierarchical model. Lancet. (2017) 390(10110):2372–81. 10.1016/S0140-6736(17)31794-4 28964589 PMC5711001

[64] Schiavo JH. PROSPERO: an international register of systematic review protocols. Med Ref Serv Q. (2019) 38(2):171–80. 10.1080/02763869.2019.1588072 31173570

[65] Shimels T Abraha M Shafie M Belay L Getnet M. Comparison of mifepristone plus misoprostol with misoprostol alone for first trimester medical abortion: a systematic review & meta-analysis protocol. EJRH. (2022) 14(2):45–51. Available at: https://ejrh.org/index.php/ejrh/article/view/396/181. (Accessed August 2022). 10.3389/fgwh.2023.1112392 PMC10038101 36970118

