# Supplementary material for: Comparison of mifepristone plus misoprostol with misoprostol alone for first trimester medical abortion: A systematic review and meta-analysis
Source: Front Glob Womens Health. 2023 Mar 6;4:1112392. doi: 10.3389/fgwh.2023.1112392 (PMC10038101; doi:10.3389/fgwh.2023.1112392)

Appendices

Appendix I: Search strategy

**Table 1**: Search strategy of PubMed for randomized clinical trial studies on ‘comparison of mifepristone plus misoprostol with misoprostol alone for first trimester medical abortion; a systematic review’

| Population | | Intervention | Comparator | Outcome | |
| --- | --- | --- | --- | --- | --- |
| Women at first trimester pregnancy | | Mifepristone plus misoprostol | Misoprostol alone | Abortion | |
| Search ((((((((((pregnant women) OR pregnant*) OR first trimester) OR first trimester) OR first trimester*) OR first trimester pregnancy) OR first trimester pregnancy) OR early pregnancy) OR early trimester pregnancy) OR first trimester gestation) OR early trimester gestation Sort by: Best Match | | Search (((((mifeprist* plus misoprost*) OR mifepristone plus misoprostol) OR (mifepristone and misoprostol)) OR mifepristone followed by misoprostol) OR mifepristone combined with misoprostol) OR mifepristone with misoprostol Sort by: Best Match | Search ((((misoprost*) OR misoprost* alone) OR misoprostol alone) OR misoprostol only) OR misoprostol Sort by: Best Match | Abortion OR termination OR medical abortion OR medical termination OR miscarriage OR pregnancy loss OR | |
| ( "Pregnancy Trimester, First/analysis"[Mesh] OR "Pregnancy Trimester, First/drug effects"[Mesh] OR "Pregnancy Trimester, First/epidemiology"[Mesh] OR "Pregnancy Trimester, First/ethnology"[Mesh] OR "Pregnancy Trimester, First/etiology"[Mesh] OR "Pregnancy Trimester, First/physiology"[Mesh] ) | | ("Mifepristone/administration and dosage"[Mesh]) OR ( "Estrenes/adverse effects"[Mesh] OR "Estrenes/etiology"[Mesh] OR "Estrenes/standards"[Mesh] OR "Estrenes/therapeutic use"[Mesh] ) | ( "Prostaglandins E, Synthetic/administration and dosage"[Mesh] OR "Prostaglandins E, Synthetic/standards"[Mesh] OR "Prostaglandins E, Synthetic/therapeutic use"[Mesh] ) | "Abortion, Induced"[Mesh] OR "Abortion, Threatened"[Mesh] OR "Abortion, Therapeutic"[Mesh] OR "Abortion, Missed"[Mesh] OR "Abortion, Incomplete"[Mesh] OR "Abortion, Habitual"[Mesh] | |
| S. no. | Search Query | | | | No. items |
| 1 | Search ((((((((((pregnant women) OR pregnant*) OR first trimester) OR first trimester) OR first trimester*) OR first trimester pregnancy) OR first trimester pregnancy) OR early pregnancy) OR early trimester pregnancy) OR first trimester gestation) OR early trimester gestation Sort by: Best Match | | | | [269113](https://www.ncbi.nlm.nih.gov/pubmed/?cmd=HistorySearch&querykey=31) |
| 2 | Search ("Pregnancy Trimester, First"[Mesh]) Sort by: Best Match | | | | [16033](https://www.ncbi.nlm.nih.gov/pubmed/?cmd=HistorySearch&querykey=32) |
| 3 | Search (((((mifeprist* plus misoprost*) OR mifepristone plus misoprostol) OR (mifepristone and misoprostol)) OR mifepristone followed by misoprostol) OR mifepristone combined with misoprostol) OR mifepristone with misoprostol Sort by: Best Match | | | | [963](https://www.ncbi.nlm.nih.gov/pubmed/?cmd=HistorySearch&querykey=33) |
| 4 | Search ("Mifepristone/administration and dosage"[Mesh]) OR ( "Estrenes/adverse effects"[Mesh] OR "Estrenes/etiology"[Mesh] OR "Estrenes/standards"[Mesh] OR "Estrenes/therapeutic use"[Mesh] ) Sort by: Best Match | | | | [16555](https://www.ncbi.nlm.nih.gov/pubmed/?cmd=HistorySearch&querykey=34) |
| 5 | Search ((((misoprost*) OR misoprost* alone) OR misoprostol alone) OR misoprostol only) OR misoprostol Sort by: Best Match | | | | [5264](https://www.ncbi.nlm.nih.gov/pubmed/?cmd=HistorySearch&querykey=35) |
| 6 | Search ( "Prostaglandins E, Synthetic/administration and dosage"[Mesh] OR "Prostaglandins E, Synthetic/standards"[Mesh] OR "Prostaglandins E, Synthetic/therapeutic use"[Mesh] ) Sort by: Best Match | | | | [3765](https://www.ncbi.nlm.nih.gov/pubmed/?cmd=HistorySearch&querykey=36) |
| 7 | Search (((((((abortion) OR termination) OR pregnancy termination) OR medical abortion) OR medical termination) OR early miscarriage) OR miscarriage) OR pregnancy loss ) OR induction) OR induc* Sort by: Best Match | | | | [3990143](https://www.ncbi.nlm.nih.gov/pubmed/?cmd=HistorySearch&querykey=37) |
| 8 | Search "Abortion, Induced"[Mesh] OR "Abortion, Threatened"[Mesh] OR "Abortion, Therapeutic"[Mesh] OR "Abortion, Missed"[Mesh] OR "Abortion, Incomplete"[Mesh] OR "Abortion, Habitual"[Mesh] Sort by: Best Match | | | | [49759](https://www.ncbi.nlm.nih.gov/pubmed/?cmd=HistorySearch&querykey=13) |
| 9 | Search quasi-randomized clinical trial Sort by: Best Match | | | | [886](https://www.ncbi.nlm.nih.gov/pubmed/?cmd=HistorySearch&querykey=19) |
| 10 | Search semi-randomized clinical trial Sort by: Best Match | | | | [32](https://www.ncbi.nlm.nih.gov/pubmed/?cmd=HistorySearch&querykey=18) |
| 11 | Search non-randomized clinical trial Sort by: Best Match | | | | [194876](https://www.ncbi.nlm.nih.gov/pubmed/?cmd=HistorySearch&querykey=17) |
| 12 | Search non-randomized control trial Sort by: Best Match | | | | [1917](https://www.ncbi.nlm.nih.gov/pubmed/?cmd=HistorySearch&querykey=16) |
| 13 | Search randomized clinical trial Sort by: Best Match | | | | [619297](https://www.ncbi.nlm.nih.gov/pubmed/?cmd=HistorySearch&querykey=14) |
| 14 | Search randomized control trial Sort by: Best Match | | | | [289106](https://www.ncbi.nlm.nih.gov/pubmed/?cmd=HistorySearch&querykey=15) |
| 15 | 1 OR 2 | | | | [269155](https://www.ncbi.nlm.nih.gov/pubmed/?cmd=HistorySearch&querykey=38) |
| 16 | 3 OR 4 | | | | [16916](https://www.ncbi.nlm.nih.gov/pubmed/?cmd=HistorySearch&querykey=39) |
| 17 | 5 OR 6 | | | | [5927](https://www.ncbi.nlm.nih.gov/pubmed/?cmd=HistorySearch&querykey=40) |
| 18 | 7 OR 8 | | | | [3990497](https://www.ncbi.nlm.nih.gov/pubmed/?cmd=HistorySearch&querykey=41) |
| 19 | 9 OR 10 OR 11 OR 12 OR 13 OR 14 | | | | [682393](https://www.ncbi.nlm.nih.gov/pubmed/?cmd=HistorySearch&querykey=20) |
| 20 | 15 AND 16 AND 17 AND 18 AND 19 (AND English AND Humans AND Abstract AND Clinical trial) | | | | [110256](https://www.ncbi.nlm.nih.gov/pubmed/?cmd=HistorySearch&querykey=46) |

**Table 2**: Search strategy and results of Cochrane CENTRAL for randomized clinical trial studies on ‘comparison of mifepristone plus misoprostol with misoprostol alone for first trimester medical abortion; a systematic review

| Search Name: Cochrane CENTRAL last search results |
| --- |
| Date Run: 20/08/2022 09:35:06 |
| Comment: Cochrane appendix |
|  |
| ID Search Hits |
| #1 mifepristone plus misoprostol 73 |
| #2 mifepristone combined with misoprostol 86 |
| #3 mifepristone and misoprostol 445 |
| #4 mifepristone with misoprostol 396 |
| #5 MeSH descriptor: [MSH Release-Inhibiting Hormone] explode all trees 7 |
| #6 MeSH descriptor: [Mifepristone] explode all trees 506 |
| #7 MeSH descriptor: [Mifepristone] explode all trees 506 |
| #8 misoprostol 3199 |
| #9 MeSH descriptor: [Misoprostol] explode all trees 1475 |
| #10 MeSH descriptor: [Misoprostol] explode all trees 1475 |
| #11 MeSH descriptor: [Misoprostol] explode all trees 1475 |
| #12 misoprostol alone 361 |
| #13 misoprostol only 497 |
| #14 first trimester 3360 |
| #15 early trimester 1553 |
| #16 first trimester pregnancy 3130 |
| #17 pregnant women 16133 |
| #18 MeSH descriptor: [Pregnancy Trimester, First] explode all trees 597 |
| #19 MeSH descriptor: [Pregnancy Trimester, First] explode all trees 597 |
| #20 MeSH descriptor: [Pregnancy Trimester, First] explode all trees 597 |
| #21 MeSH descriptor: [Pregnancy Trimester, First] explode all trees 597 |
| #22 abortion 5207 |
| #23 medical abortion 1876 |
| #24 medication abortion 327 |
| #25 complete abortion 711 |
| #26 MeSH descriptor: [Abortion, Induced] explode all trees 1036 |
| #27 MeSH descriptor: [Abortion Applicants] explode all trees 2 |
| #28 MeSH descriptor: [Abortion Applicants] explode all trees 2 |
| #29 1 OR 2 OR 3 OR 4 1464607 |
| #30 5 OR 6 OR 7 1034080 |
| #31 9 OR 10 OR 11 1007423 |
| #32 30 AND 31 219732 |
| #33 29 OR 32 211984 |
| #34 8 OR 12 OR 13 751769 |
| #35 31 OR 34 1229484 |
| #36 14 OR 15 OR 16 OR 17 599461 |
| #37 18 OR 19 OR 20 OR 21 622608 |
| #38 36 OR 37 203223 |
| #39 22 OR 23 OR 24 OR 25 556761 |
| #40 26 OR 27 OR 28 359382 |
| #41 39 OR 40 236519 |
| #42 33 AND 35 AND 38 AND 41 in Trials with 'Cochrane Brazil', 'Cochrane South Asia', 'Pregnancy and Childbirth', 'Cochrane Germany', 'Fertility Regulation', 'Gynaecology and Fertility', 'Cochrane UK', 'Cochrane Australia', 'Cochrane Thailand', 'Cochrane Canada' in Cochrane Groups 15 |

**Table 3**: Search strategy and results of EMBASE (Ovid) for randomized clinical trial studies on ‘comparison of mifepristone plus misoprostol with misoprostol alone for first trimester medical abortion; a systematic review

| **Embase** |
| --- |
| **Session Results** |
| ....................................................... |
| No. Query Results Results Date |
| #3. #1 OR #2 3 20 Aug 2022 |
| #2. ('controlled study'/de OR 'human'/de OR 'human 1 20 Aug 2022 |
| experiment'/de OR 'randomized controlled |
| trial'/de) AND ('incomplete abortion'/dm OR |
| 'missed abortion'/dm OR 'muscle cramp'/dm OR |
| 'nausea'/dm OR 'retained placenta'/dm) AND |
| ('archives of gynecology and obstetrics'/jt OR |
| 'contraception'/jt) AND ('adverse drug |
| reaction'/lnk OR 'buccal drug administration'/lnk |
| OR 'drug combination'/lnk OR 'drug |
| comparison'/lnk OR 'side effect'/lnk) AND |
| 'article'/it AND [embryo]/lim AND |
| ('mifepristone'/dd OR 'misoprostol'/dd) |
| #1. ('pregnant woman'/exp OR 'first trimester 2 20 Aug 2022 |
| pregnancy'/exp OR 'early pregnancy' OR 'first |
| trimester' OR 'first trimester pregnancy' OR |
| 'pregnancy trimester, first' OR 'pregnancy, |
| early' OR 'pregnancy, first trimester') AND |
| ('mifepristone plus misoprostol'/exp OR 'medabon' |
| OR 'mifegyne combikit' OR 'mifepristone plus |

Appendix II: Data extraction instrument

#Only append the JBI or non-JBI data extraction instrument if the standardized tool has been modified in any way, otherwise simply cite the tool used in the text. Any modifications made to the instrument should also be described in the text.#

Appendix III: Studies excluded

Bracken H, Zuberi N, de Guevara Puerto AL, Mayi-Tsonga S, Buendía Gómez M, Irfan Ahmed S, et al. Mifepristone and sublingual misoprostol versus sublingual misoprostol alone for missed abortion: Results of a randomized placebo-controlled trial. Contraception. 2019;99(5):315–6.

*Reason for exclusion: Not the population of interest to this review*

Dabash R, Blum J, Raghavan S, Ngoc NTN, Chelli H, Hajri S, et al. Outcomes of a double-blind randomized trial comparing misoprostol-only to mifepristone+misoprostol for home-based early medical abortion. International journal of gynaecology and obstetrics. 2012;119:S315.

*Reason for exclusion: Study is a duplicate of same study by Ngoc eta al., 2011*

Dahiya K. Randomized trial of mifepristone and buccal misoprostol vs misoprostol alone for medical abortion. International journal of gynecology and obstetrics. 2015;131:E589.

*Reason for exclusion: A sole author duplicate of an earlier study (Dahiya et al. 2012)*

Ngo TD, Park MH. Mifepristone+misoprostol vs. misoprostol alone for early medical abortion. Contraception. 2012;85(2):219; author reply 219-20.

*Reason for exclusion: Study is a duplicate of Ngoc et al., 2011*

Ngoc N, Blum J, Nga N, Raghavan S, Winikoff B. Medical abortion with misoprostol only versus mifepristone plus misoprostol: results from a randomized controlled trial. International journal of gynaecology and obstetrics. 2009;107:S286‐.

*Reason for exclusion: Not population of interest.*

Blum J, Ngoc NT, Nga NT, Raghavan S, Winikoff B. Medical abortion with misoprostol only vs. mifepristone plus misoprostol: results from a randomized controlled trial. Contraception. 2009;80(2):195‐.

*Reason for Exclusion: Unable to access full text.*

Chai J, Wong CY, Ho PC. A randomized clinical trial comparing the short-term side effects of sublingual and buccal routes of misoprostol administration for medical abortions up to 63 days' gestation. Contraception. 2013;87(4):480-5.

*Reason for exclusion: Not comparator and outcome of interest*.

Chong E, Tsereteli T, Nguyen NN, Winikoff B. A randomized controlled trial of different buccal misoprostol doses in mifepristone medical abortion. Contraception. 2012;86(3):251-6.

*Reason for exclusion: Not comparator of interest.*

Coyaji K, Krishna U, Ambardekar S, Bracken H, Raote V, Mandlekar A, et al. Are two doses of misoprostol after mifepristone for early abortion better than one? Bjog. 2007;114(3):271-8.

*Reason for exclusion: Not comparator of interest.*

Creinin MD, Schwartz JL, Pymar HC, Fink W. Efficacy of mifepristone followed on the same day by misoprostol for early termination of pregnancy: report of a randomised trial. Bjog. 2001;108(5):469-73.

*Reason for exclusion: Not comparator of interest.*

el-Refaey H, Rajasekar D, Abdalla M, Calder L, Templeton A. Induction of abortion with mifepristone (RU 486) and oral or vaginal misoprostol. The New England journal of medicine. 1995;332(15):983-7.

*Reason for exclusion: Not comparator of interest.*

Goel A, Mittal S, Taneja BK, Singal N, Attri S. Simultaneous administration of mifepristone and misoprostol for early termination of pregnancy: a randomized controlled trial. Archives of gynecology and obstetrics. 2011;283(6):1409-13.

Hamoda H, Ashok PW, Flett GM, Templeton A. A randomised controlled trial of mifepristone in combination with misoprostol administered sublingually or vaginally for medical abortion up to 13 weeks of gestation. Bjog. 2005;112(8):1102-8.

*Reason for exclusion: Not comparator of interest.*

Li CL, Chen DJ, Song LP, Wang Y, Zhang ZF, Liu MX, et al. Effectiveness and Safety of Lower Doses of Mifepristone Combined With Misoprostol for the Termination of Ultra-Early Pregnancy: A Dose-Ranging Randomized Controlled Trial. Reproductive sciences (Thousand Oaks, Calif). 2015;22(6):706-11.

*Reason for exclusion: Not comparator of interest.*

Liao AH, Han XJ, Wu SY, Xiao D, Xiong CL, Wu XR. Randomized, double-blind, controlled trial of mifepristone in capsule versus tablet form followed by misoprostol for early medical abortion. European journal of obstetrics, gynecology, and reproductive biology. 2004;116(2):211-6.

*Reason for exclusion: Not comparator of interest.*

Mittal S, Agarwal S, Kumar S, Batra A. Comparison or oral versus vaginal misoprostol & continued use of misoprostol after mifepristone for early medical abortion. The Indian journal of medical research. 2005;122(2):132-6.

*Reason for exclusion: Not comparator of interest.*

Raghavan S, Maistruk G, Shochet T, Bannikov V, Posohova S, Zhuk S, et al. Efficacy and acceptability of early mifepristone-misoprostol medical abortion in Ukraine: results of two clinical trials. The European journal of contraception & reproductive health care : the official journal of the European Society of Contraception. 2013;18(2):112-9.

*Reason for exclusion: Not comparator of interest.*

Reeves MF, Kudva A, Creinin MD. Medical abortion outcomes after a second dose of misoprostol for persistent gestational sac. Contraception. 2008;78(4):332-5.

*Reason for exclusion: Not comparator of interest.*

Reeves MF, Monmaney JA, Creinin MD. Predictors of uterine evacuation following early medical abortion with mifepristone and misoprostol. Contraception. 2016;93(2):119-25.

*Reason for exclusion: Not comparator of interest.*

Schaff EA, Fielding SL, Westhoff C. Randomized trial of oral versus vaginal misoprostol at one day after mifepristone for early medical abortion. Contraception. 2001;64(2):81-5.

*Reason for exclusion: Not comparator of interest.*

Shannon C, Wiebe E, Jacot F, Guilbert E, Dunn S, Sheldon WR, et al. Regimens of misoprostol with mifepristone for early medical abortion: a randomised trial. Bjog. 2006;113(6):621-8.

*Reason for exclusion: Not comparator of interest.*

Tang OS, Chan CC, Ng EH, Lee SW, Ho PC. A prospective, randomized, placebo-controlled trial on the use of mifepristone with sublingual or vaginal misoprostol for medical abortions of less than 9 weeks gestation. Human reproduction (Oxford, England). 2003;18(11):2315-8.

*Reason for exclusion: Not comparator of interest.*

Appendix IV: Characteristics of Included Studies

*Table: Characteristics of Included Studies - Randomized Controlled Trial Form*

| **Study** | **Country** | **Setting/context** | **Participant characteristics** | **Groups** | **Outcomes measured** | **Description of main results** |
| --- | --- | --- | --- | --- | --- | --- |
| Chawdhary R, Rana A, Pradhan N. 2009. | Nepal | University teaching hospital | Women demonstrating an (intact) single intrauterine pregnancy (IUP) up to a 63-day period of gestation (POG) | Group A (mifepristone + misoprostol) received mifepristone 200 mg orally (n=50)Group B (Misoprostol only) had an insertion of saline-soaked misoprostol 800 mg in the posterior vaginal fornix after thoroughly cleaning the vagina with dry cotton (to remove TVS gel) after which they were instructed to remain in a supine for atleast 15 minutes (n=50) | The primary outcome measure was complete abortion, which was obtained by the history of stoppage of pain and bleeding after the complete expulsion of fetus enclosed in the sac; a clinical finding of a completely closed cervical Os, without further evidence of bleeding; and the depiction of an empty uterine cavity on TVS | In group A complete abortion occurred in 47 (94%) of the women and, In group B, the total number of complete abortion became 43 (86%) |
| Blum J, Raghavan S, Dabash R, Ngoc NTN, Chelli H, Hajri S, et al. 2012. | Tunisia and Vietnam | Participants were recruited from large maternity hospitals and, were allowed to either live or work within a reasonable distance from the hospitals | Pregnant women presenting for first trimester medical abortion | Group one comprised of women who recived the combined mifepristone–misoprostol regimen (n=220)Group two women recieved the mifepristone-only regimen (n=221) | The primary outcome measure was complete uterine evacuation without surgical evacuation for any reason. | Higher proportion of women in the mifepristone–misoprostol group (n=195; 92.9%) experienced complete abortion without surgical evacuation compared with the misoprostol-only group (n=170; 78.0%). The RR was 0.84 (95% CI, 0.78–0.91; Pb0.001) |
| Dalenda C, Ines N, Fathia B, Malika A, Bechir Z, Ezzeddine S, et al. 2010. | Tunisia | Obsterics and Gynecology Center of Maternity | Women seeking medical abortion at 9 to 12 weeks of gestation. | Seventy-three patients were given a fixed protocol of 200 mg of mifepristone followed 48 h later by 400 mcg oral misoprostol (Group 1) (n=73)A second group of 49 patients was administered 800-mcg intravaginal single-dose misoprostol (Group 2) (n=49) | This study sought to compare safety, efficacy and acceptability of these two nonsurgical abortion regimens. | Fifty-nine (80.8%) women in Group 1 had complete abortion vs. 38 (77.4%) women in Group 2 (p=.66). Abdominal pain was observed significantly more often in Group 2 (35/49 (71.4%) vs. 32/73 (43.8%) in Group 1, pb.0001. Medical abortion was equally acceptable among the two groups [37/49 (75.5%) and 55/73 (75.7%), p=.89]. |
| Fekih M, Fathallah K, Ben Regaya L, Bouguizane S, Chaieb A, Bibi M, et al. 2010. | Tunisia | Farhat Hached Teaching Hospital | healthy pregnant women requesting medical abortion in the first trimester (up to 56 days) | Women in group 1 took 200 mg of oral mifepristone in the hospital, followed by 400 μg of oral misoprostol (Cytotec; Pfizer, France) 48 hours later at home if abortion had not occured (n=126)Women in group 2 were administeredwith 800 μg of sublingual misoprostol (Cytotec) in the hospital at about 8:00AM and then discharged home. | The primary outcome measure was the mean drop in hematocrit and, secondary outcome measures were decrease in hemoglobin level, duration of bleeding, recourse to uterotonics, and expulsion time. | Complete abortion rates at the 2-week follow-up were similar in groups 1 and 2 (94.5% vs 92.1%, respectively; P=0.3). Following administration of misoprostol, at least one adverse effect was reported by 103 (81.7%) women in group 1 compared with 100 (79.4%)women in group 2. Overall, 96% of women in group 1 and 92% of women in group 2 reported being satisfied with the treatment (P=0.08). |
| Jain JK, Dutton C, Harwood B, Meckstroth KR, Mishell DR. 2002. | USA | Women's and Children's Hospital and Affiliated Clinics | Healthy women desiring termination of pregnancies ≤56 days gestation. | Women assigned to group 1 recieved mifepristone on study day 1, followed 48 hours later ,by 800micg vaginal misoprostol.Women assigned to group 2 recieved placebo on day 1, followed 48 hours latter by 800micg misoprostol. | Abortion success was defined as complete abortion without the use of surgical aspiration. | Successful medical abortions occurred in 114 out of 119 subjects (95.7%) after mifepristone followed by vaginal misoprostol. In all, 110 out of 125 subjects (88.0%) successfully aborted after placebo and vaginal misoprostol. |
| Dahiya K, Ahuja K, Dhingra A, Duhan N, Nanda S. 2012. | India | Teaching hospital | Pregnant women with gestational age of 56 days or less | In group A, patients received 200 mg mifepristone on day 1, followed by buccal misoprostol 800 µg on day 2 (n=50)In group B, patients received 800 µg buccal misoprostol only on day 1 (n=50) | Complete abortion was the principal outcome measure. Secondary outcome measures were side-effects and acceptability | Forty-six (92%) patients in group A and 37 (74%) patients in group B aborted successfully (p value 0.017). In group B, three (6%) patients had missed abortion and two (4%) patients had continued pregnancy whereas none of the patients in group A had missed abortion or continued pregnancy. The overall method acceptance was 100% whereas the overall route acceptance was 83%. |
| Ngoc NTN, Blum J, Raghavan S, Nga NTB, Dabash R, Diop A, et al. 2011. | Vietnam | Tertiary Hospital | Pregnant mothers with <63 days of gestation | Group 1 recieved two doses of 800 mcg buccal misoprostol 24 h apart (n=198)Group 2 recieved 200 mg mifepristone and 800 mcg buccal misoprostol 24 h (n=202) | complete abortion, ongoing pregnancy and side effects. | Complete abortion occurred for 76.2% (n=147) of women allocated to misoprostol-only vs. 96.5% (n=194) of those given mifepristone+misoprostol (RR 0.79, 95% CI 0.73–0.86). Ongoing pregnancy was documented for 16.6% (32) of misoprostol-only users and 1.5% (3) of mifepristone +misoprostol users (1.62, 0.68–3.90). Side effects were generally similar for both groups, although significantly more women allocated to misoprostol-only reported diarrhea. |
| Schreiber CA, Creinin MD, Atrio J, Sonalkar S, Ratcliffe SJ, Barnhart KT. 2018. | USA | Hospital | Healthy women 18 years of age or older and had an ultrasound examination that showed a nonviable intrauterine pregnancy between 5 and 12 completed weeks of gestation | Group 1 recieved 200 mg of mifepristone, administered orally, followed by 800 μg of misoprostol, administered vaginally approximately 24 hours later (mifepristone-pretreatment group)Group 2 recieved standard therapy with 800 μg of misoprostol alone, administered vaginally (misoprostol-alone group), on trial day 1 | Main outcomes were; complete expulsion and incomplete abortion. Associated side effects evaluated in both groups included; bleeding, infection, fatigue, headache, chills, nausea and vomiting, diarrhea, cramping and fever. | Complete expulsion after one dose of misoprostol occurred in 124 of 148 women in the mifepristone-pretreatment group and in 100 of 149 women in the misoprostol-alone group. Bleeding that resulted in blood transfusion occurred in 2.0% of the women in the mifepristone-pretreatment group and in 0.7% of the women in the misoprostol-alone group; pelvic infection was diagnosed in 1.3% of the women in each group. |
| Stockheim D, Machtinger R, Wiser A, Dulitzky M, Soriano D, Goldenberg M, et al. 2006. | Israel | University affiliated medical center | 115 women with blighted ovum or missed abortion and less 9 weeks of gestation were included | Group 1 patients received orally 600 mg mifepristone followed, 48 hours later by misoprostol (n=58)Group 2 patients recieved 800mcg misoprostol only regimen and 48 hours latter an other 800mcg of the drug (n=57) | Failure was defined as surgical intervention due to retained gestational sac 48 hours after completion of the drug protocol, severe symptoms, or suspected retained products of conception after the menstrual period. | The success rate was similar in groups I and II: 38 of 58 patients (65.5%) versus 42 of 57 patients (73.6%), respectively. No cases of severe infection or bleeding necessitating blood transfusion occurred. |

### Funnel plots for publication bias


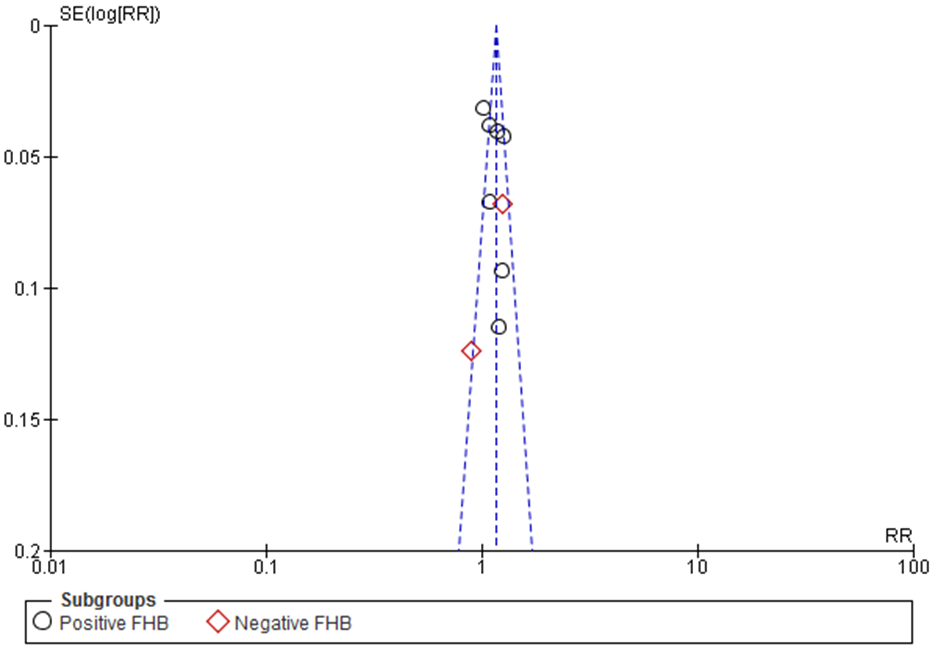


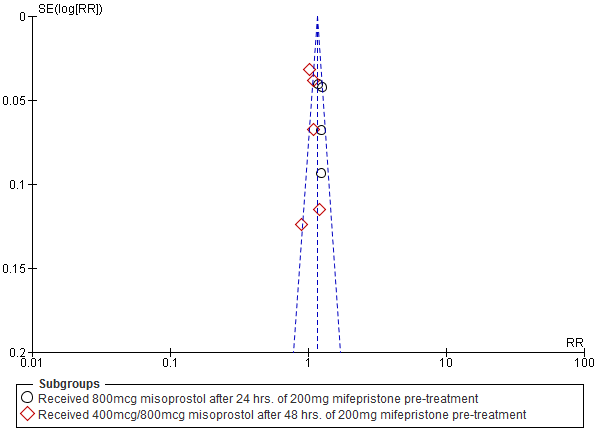


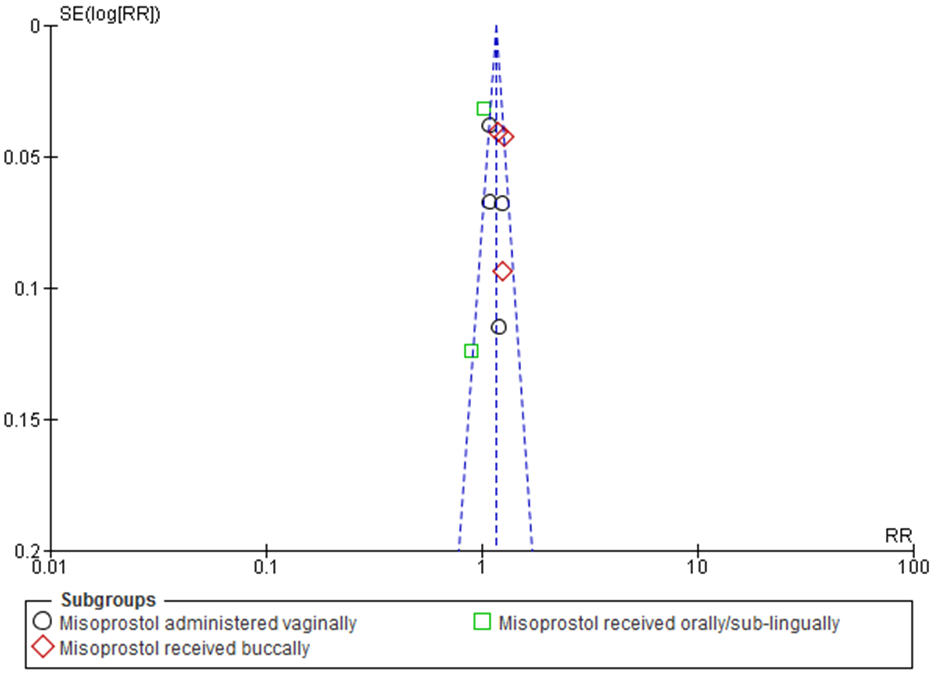

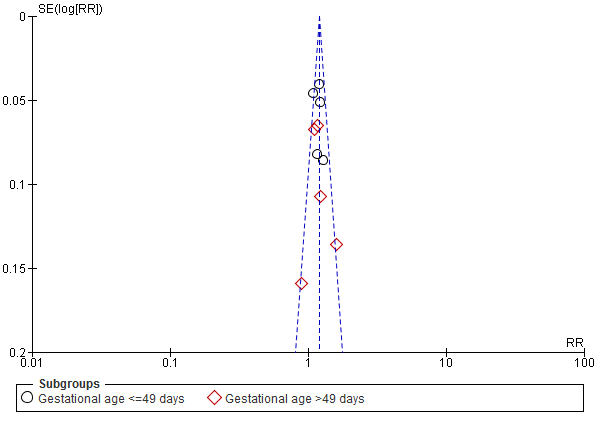

Supplement: Supplementary file 1 [file Table1.docx]
